# Impact of Quercetin on the Functional and Structural Properties of Chitooligosaccharide (CHOS)-Enriched Surimi

**DOI:** 10.3390/gels12090776

**Published:** 2026-08-29

**Authors:** Bikash Kumar Pati, M. Bhargavi Priyadarshini, Naresh Kumar Mehta, Arun Bhai Patel, Kumar Gaurav, Shreya Anand

**Affiliations:** 1 College of Fisheries, Odisha University of Agriculture and Technology, Berhampur 760007, Odisha, India; bkpati@ouat.ac.in; 2 College of Fisheries, Central Agricultural University, Agartala 799210, Tripura, India; nareshfishco@gmail.com (N.K.M.); arun.b.patel@gmail.com (A.B.P.); kgcof201921@gmail.com (K.G.); shreyaanand009@gmail.com (S.A.)

**Keywords:** pangasius surimi, quercetin, chitooligosaccharides, gel strength, microstructure, antioxidant activity, water-holding capacity

## Abstract

This study evaluated the impact of varying quercetin (Q; 0.01–0.05%) incorporations on the quality, functional, structural, and bio-functional properties of CHOS-enriched (0.75%; *w*/*w*) pangasius surimi gel. Gel samples were analyzed for gel strength, texture profile, colour, water-holding capacity (WHC), protein interactions, structural changes, thermal stability, antioxidant/antimicrobial activities, and sensory parameters. Results indicated that Q significantly decreased pH while exerting a minor effect on proximate composition. Incorporation of Q up to 0.04% (Q4) remarkably improved breaking force (654.70 g), deformation (1.13 cm), gel strength (707.82 g cm), hardness, and WHC (90.66%), while reducing expressible moisture content and proteolysis. Molecular interactions, FT-IR spectra, SDS-PAGE, and SEM confirmed that Q promoted cross-linking of myosin heavy chains via hydrogen bonding and hydrophobic interactions, creating a denser, more compact gel matrix with higher thermal stability. Additionally, Q addition significantly enhanced 2,2′-azino-bis (3-ethylbenzothiazoline-6-sulfonic acid) (ABTS)/2,2-diphenyl-1-picrylhydrazyl (DPPH) radical scavenging and antibacterial activity against *Escherichia coli* (*E. coli*) and *Staphylococcus aureus* (*S. aureus*) in a concentration-dependent manner, though lightness and whiteness indices decreased due to Q’s natural yellow pigment.

## 1. Introduction

Surimi serves as an intermediate ingredient that facilitates the large-scale production of restructured seafood products, such as crab-flavoured sticks and fish cakes, with consistent and controllable quality [1,2]. Common freshwater fish used for surimi production include silver carp, grass carp, snakehead, and pangasius [3]. Amongst these, pangasius (*Pangasianodon hypophthalmus*) fish are valued for their nutritional content and affordability, and their taste is comparable to that of other widely consumed whitefish species [4]. India is currently the world’s second-largest producer of pangasius, with an estimated production of 450,000 tons in 2024, primarily from Andhra Pradesh and West Bengal states. The majority of this production is consumed domestically, while a small but increasing volume of frozen fillets is exported, particularly to the Middle East [5]. The abundant availability and cost-effectiveness of pangasius make it an attractive raw material for surimi production, particularly given the decline in marine fish resources.

Surimi is manufactured into a diverse range of products: frozen surimi blocks, kamaboko-type heat-set gels, fish cakes, fish balls, and crab-flavoured sticks, and the storage behaviour of each differs markedly depending on its processing history, moisture content, and packaging [6]. Frozen surimi blocks, the primary raw material, are protected against myofibrillar protein denaturation by the addition of cryoprotectants such as sucrose and sorbitol, yet even under frozen storage, protein denaturation, lipid oxidation, and moisture migration proceed gradually, causing progressive declines in GS, WHC, and whiteness. Cooked gel products, in contrast, are stored under refrigeration, where their quality is governed largely by post-process contamination, outgrowth of psychrotrophic spoilage bacteria, and residual endogenous enzyme activity [7]. In the absence of any preservative or antimicrobial treatment, refrigerated cooked surimi gels undergo systematic biochemical deterioration; free-water content, pH, hardness, total volatile basic nitrogen (TVBN), thiobarbituric acid-reactive substances (TBARS), and total plate counts (TPC) all increase progressively, while whiteness, springiness, cohesiveness, protein solubility, WHC, and total sulfhydryl content (TSC) decline, accompanied by continuous protein degradation, as noticed in cooked silver carp surimi batter [8] and in tilapia surimi during chilled storage [9]. In pangasius surimi-based cooked analogues stored at 3 ± 1 °C, the pH of the control correlated positively with TVBN and peroxide value (PV); psychrophilic microorganisms were detected from day 9 onward, and TVBN reached 24.65 mg N % by day 21 [10], confirming that ready-to-cook surimi gels from this species are highly susceptible to early microbial and biochemical spoilage. Sensorially, these biochemical changes manifest as off-odours, off-flavours, drip loss, and textural softening, culminating in consumer rejection if no intervention is applied. The rate and pattern of these changes proximate, microbial, biochemical, and sensory therefore differ substantially between raw frozen blocks, chilled cooked gels, and ready-to-eat products since each presents a distinct matrix, water activity, and thermal history, and this variability must be considered when evaluating the protective efficacy of any added functional ingredient.

Chitooligosaccharides (CHOS) are cationic oligosaccharides composed mainly of glucosamine and N-acetylglucosamine units joined by β-(1,4) glycosidic bonds, with a typical degree of polymerization from 2 to 10 [11]. Previous studies have demonstrated that the incorporation of 10 g/kg CHOS derived from squid pen significantly improved the gel properties of sardine surimi [12]. More recently, snow crab shell-derived CHOS was reported to improve the textural properties of pangasius surimi [13], highlighting its potential as a functional ingredient in freshwater surimi products. To further enhance the functional properties of CHOS, several studies have conjugated or grafted phenolic compounds onto the CHOS backbone. A CHOS-epigallocatechin gallate (EGCG) conjugate significantly prolongs the shelf life of Asian green mussels by inhibiting microbial growth. Its antimicrobial activity involves disruption of bacterial cell walls and interaction with negatively charged membrane components, resulting in increased membrane permeability, cell leakage, and bacterial death [14]. Likewise, CHOS-gallic acid exhibited strong antioxidant and water-retaining properties, thereby stabilizing shrimp muscle during long-term frozen storage [15].

Q, a natural flavonoid with antimicrobial and antioxidant activities, could interact electrostatically with myofibrillar proteins, thereby preserving the α-helical structure and enhancing the functional properties of shrimp surimi [16]. Although CHOS-phenolic conjugates have shown promising functional benefits, their synthesis generally involves laborious, multi-step procedures requiring chemical activation, catalysis, controlled reaction conditions, and post-synthesis purification, which escalate production costs and limit industrial scalability [17,18]. Recent conjugation approaches, activated ester modification, enzyme-mediated grafting, and free radical-induced coupling, each proceed through process-controlled stages; for example, grafting Q onto chitosan via a covalent method requires pre-functionalization of chitosan with reagents such as ethyl chloroformate to amplify amine reactivity toward the hydroxyl groups of Q [19], followed by grafting and recovery steps. Moreover, previous applications of such conjugates have largely targeted the shelf-life extension of mussels and shrimp, whereas systematic evidence for their effects on gel network formation, textural properties, and thermal stability in freshwater fish surimi remains scarce. Pangasius surimi suffers from rapid pH shifts, early psychrophilic microbial outgrowth, and progressive loss of texture and whiteness during storage, as outlined above, yet no study has so far examined whether the direct incorporation of a low-cost phenolic compound such as Q into CHOS-fortified pangasius surimi can replicate the functional benefits of conjugated systems, an important gap, since phenolic compounds can either promote or impair protein gel network formation, depending on their concentration and mode of interaction with the myofibrillar protein matrix. Therefore, instead of preparing CHOS-Q conjugates, the present study investigated the direct incorporation of Q into pangasius surimi that was pre-fortified with snow crab shell-derived CHOS as a simpler and more practical strategy, with the hypothesis that the combined actions of CHOS and Q would synergistically retard spoilage and reinforce the gel network. Hence, the effects of different Q concentrations (0.01–0.05%) on the physicochemical, gel, textural, structural, thermal, antioxidant, and antimicrobial properties of CHOS-enriched pangasius surimi gel are systematically examined in the present report.

## 2. Results and Discussion

### 2.1. Effect of Different Levels of Q on CHOS-Enriched Pangasius Surimi Gel

#### 2.1.1. Proximate Composition and pH

The proximate composition of CHOS-enriched (0.75%; *w*/*w*) pangasius surimi gel treated with different levels of Q is given in Table 1. The incorporation of Q had a minimal impact on the proximate composition of the CHOS-enriched pangasius surimi gel, suggesting that the fundamental composition of the gel matrix remained relatively consistent across treatments; the moisture content remained within a close range (81.29% to 81.53%), while the protein content was significantly increased from 12.61% in Q1 to 13.70% in Q5. Furthermore, the fat content exhibited a notable change from 0.63% (Q2) to 1.35% (Q1), and the ash content changed slightly. The present CHOS-enriched gel system had relatively lower moisture and protein contents, along with lower fat levels than the conventional pangasius surimi prepared by multiple washing cycles, but possessed a visibly higher ash content. This variation is expected since the current formulation contains CHOS, which makes its composition different from normal washed surimi. Previous reports on pangasius surimi showed a moisture content ranging from 84.26 to 85.05%, protein content from 12.93 to 15.94%, fat content from 1.77 to 3.75%, and ash content from 0.20 to 0.39% [20]. The pH of CHOS-enriched pangasius surimi gel showed a significant decreasing trend with increasing levels of Q incorporation (*p* < 0.05). The control sample exhibited the highest pH value (6.88), while Q-treated samples showed progressively lower pH values, ranging from 6.78 in Q1 to 6.53 in Q5. Among the treatments, Q5 recorded the lowest pH, followed by Q4 (6.57) and Q3 (6.66). Q is a polyphenolic flavonoid containing multiple hydroxyl (-OH) groups that can release hydrogen ions (H^+^) [21] in the meat system, thereby lowering the pH. The high-protein-content food exhibits strong buffering capacity; however, the interaction of Q with protein molecules might have diminished the buffering capacity of the surimi matrix, resulting in a lower pH [22]. The result suggests that Q mainly affected the acidity of the system while having a limited effect on the proximate composition.

#### 2.1.2. Gel Properties

The effects of Q at varying concentrations (0.01–0.05%) on gel properties are presented in Table 2. The incorporation of Q significantly improved the gel properties of the pangasius surimi gel. The breaking force (BF) was enhanced progressively from Q1 (0.01%) to Q4 (0.04%), reaching the maximum value at Q4 (654.7 g), which was significantly higher than the control (*p* < 0.05). However, a marginal decrease was observed at the highest concentration (Q5, 0.05%). The deformation (DF) values also increased with the concomitant addition of Q. The control exhibited the lowest DF (0.91 cm), while Q4 showed the highest DF (1.13 cm). The highest gel strength (GS) was observed in Q4 (707.82 g cm), which was significantly higher than all other treatments (*p* < 0.05). However, a marginal decline at 0.05% suggests that excessive Q may disrupt optimal gel network formation. The observed improvements in gel properties could be attributed to enhanced intermolecular interactions between Q and myosin. Chen et al. [23] recorded that the myosin-Q complex showed a significantly higher hydrogen bond (H-bond) content than the other treatment groups (flavonoids), emphasizing the important contribution of flavonol phenolic hydroxyl groups to H-bond formation within the myosin gel matrix. These hydroxyl groups readily interact with polar residues on myosin molecules, thereby strengthening intermolecular H-bonding. Consequently, a denser, more uniform, and less porous gel network is formed [23], which supports the enhanced GS observed in the present study. Consistent with these findings, Li et al. [16] demonstrated that Q incorporation (60 μmol phenolic/g myofibrillar protein) increased shrimp surimi GS by 2.53-fold, attributing this effect to H-bonding between polyphenol hydroxyl groups and hydrogen acceptors on protein chains. However, excessive polyphenol addition can have adverse effects. Buamard et al. [24] suggested that high concentrations of phenolic compounds may induce excessive cross-linking, leading to protein aggregation and coagulation, thereby producing a disordered gel network. Similarly, Li et al. [25] observed that increasing phenolic hydroxyl concentrations from EGCG, catechin, and tannic acid negatively affected silver carp surimi gel properties due to excessive and rapid protein aggregation. This phenomenon may occur when protein denaturation proceeds faster than orderly aggregation, ultimately weakening the gel structure. Similar to the present findings, Guo [26] demonstrated that the molecular size and structural complexity of phenolic compounds determine their effects on myofibrillar protein gelation. Under mild oxidative conditions, low-molecular-weight phenolic acids (6–60 μmol/g protein) increased GS and BF by up to 86% and 53%, respectively, whereas larger polyphenols, such as EGCG, promoted excessive aggregation that weakened gel formation [27]. A slower and more controlled aggregation process favours the development of a stable and highly viscoelastic protein network, whereas rapid aggregation disrupts the structural organization of myofibrillar proteins [27]. Recent evidence has further suggested that natural polyphenolic additives exhibit a concentration-dependent response, either strengthening or destabilizing the gel network depending on their dosage and interaction with muscle proteins [28]. In agreement with these findings, Q supplementation up to 0.04% significantly improved the GS and textural properties of CHOS-enriched pangasius surimi gel, whereas a slight decline at 0.05% indicated a transition from beneficial protein cross-linking to excessive aggregation. Therefore, 0.04% Q may be considered the optimum concentration for improving the structural and textural properties of the surimi gel system [9,29,30].

#### 2.1.3. Texture Profile Analysis (TPA)

The TPA parameters of pangasius surimi gel enriched with CHOS at different levels of Q are presented in Table 3. The TPA of CHOS-enriched pangasius surimi gel showed significant improvements (*p* < 0.05) with increasing Q levels up to Q4. Hardness, springiness, cohesiveness, chewiness, and resilience increased progressively and reached their highest values in Q4 (8484.51 g, 0.96, 0.83, 5509.15 g cm, and 0.52, respectively). However, a marginal decline in these parameters was observed in Q5. The enhancement in TPA properties up to Q4 may be associated with protein–polyphenol interactions, which promoted cross-linking within the myofibrillar proteins, resulting in a stronger, denser, and more stable gel network [31]. In contrast, the reduction in texture parameters at a higher Q concentration (Q5) could be due to excessive cross-linking, leading to protein coagulation and self-aggregation of phenolic compounds, thereby hindering the formation of an ordered gel matrix and negatively affecting gel properties [31]. Likewise, a comparable result was observed in Indian mackerel mince gel incorporated with *Duea ching* (*Ficus botryocarpa* Miq.), which contains quercetin-3-galactoside in combination with microbial transglutaminase [32]. More studies further support the dose-dependent modulation of texture parameters in fish protein gel systems. Lv et al. [33] reported that polyphenols such as dihydroquercetin, a structural analogue of Q, increased the WHC, GS, and textural properties of *Penaeus vannamei* myosin gels after freezing. Furthermore, SEM revealed smoother and more compact gel surfaces in the polyphenol-treated groups than in the control. Similarly, Tian et al. [34] demonstrated that the addition of EGCG at concentrations of 0.01–0.02% effectively inhibited the decline in texture and GS of silver carp surimi during repeated freeze–thaw cycles. However, excessive EGCG supplementation (0.03%) resulted in a loose protein network and increased moisture loss. These findings are consistent with the present study, in which all texture profile analysis parameters improved progressively up to Q4 and declined when the Q concentration reached 0.05%, indicating that optimum polyphenol supplementation promotes protein network stabilization, whereas excessive concentrations adversely affect the gel structure.

#### 2.1.4. Instrumental Colour Attributes and Whiteness

Table 4 shows the effects of different levels of Q on instrumental colour attributes and whiteness of CHOS-enriched pangasius surimi gel. The instrumental colour of the CHOS-enriched surimi gel was significantly altered by the incorporation of Q (*p* < 0.05). The lightness (L*) value decreased significantly from 81.03 (control) to 71.91 (Q5), and the whiteness index also decreased significantly from 74.97 (control) to 62.58 (Q5) (*p* < 0.05). This reduction in brightness is common when adding non-proteinaceous additives to surimi, as they influence the reflection and absorption of light by the protein network [20]. The significant increase in yellowness (b*) from 16.35 (control) to 26.73 (Q5) is a direct consequence of the natural yellow pigment of Q. This behaviour was consistent with a previous study, where Q was added to fish oil-enriched gels, resulting in a substantial increase in yellowness [35]. Sharma et al. [36] reported that silver carp surimi gels fortified with pineapple peel extract, a polyphenol-rich source, displayed a slight but consistent reduction in whiteness, even at the optimal 1% level that maximized GS (504.13 g cm) and BF (511.64 g). Likewise, Tan et al. [37] observed that the whiteness of mackerel puree declined during refrigerated storage, although the addition of tea polyphenols minimized colour changes while preserving a compact gel microstructure. Additionally, green tea extract lowered the redness (a*) values of tilapia mechanically separated meat throughout 180 days of frozen storage [38], reflecting the pigment–protein interactions that drive the greenness shift observed in the present gels. Other studies on polyphenolic fortification have noted that while functional properties may improve, the whiteness index often suffers significantly once the concentration exceeds the optimum level [30]. Together, these reports confirm that even moderate polyphenol incorporation partially reduces the whiteness of the gel and raises yellowness, supporting the present observation that a decrease in whiteness is an inherent, concentration-dependent consequence of Q fortification in CHOS-enriched pangasius surimi. In the context of surimi, which is valued for its white appearance, these changes are often seen as a technological adjustment.

#### 2.1.5. Expressible Moisture Content (EMC) and Water-Holding Capacity (WHC)

**Figure 1:**
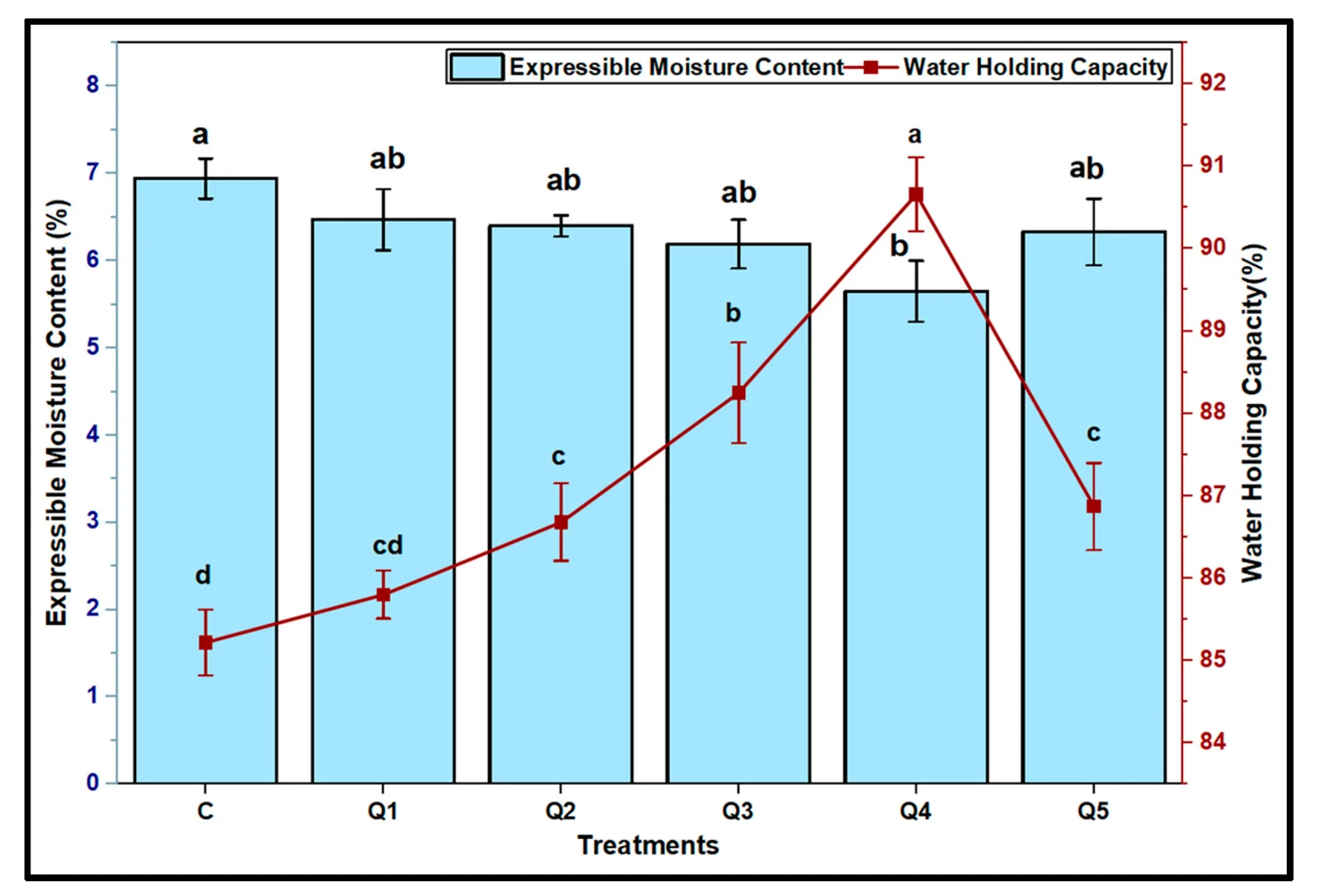
Effects of different levels of Q on EMC and WHC of CHOS-enriched pangasius surimi gel. Values are expressed as means ± standard deviations (n = 3). Different letters on the symbols indicate that the values are significantly different (*p* ˂ 0.05) between the treatments. CHOS-enriched pangasius surimi gel without Q (C); CHOS-enriched pangasius surimi gel with 0.01% Q (Q1); CHOS-enriched pangasius surimi gel with 0.02% Q (Q2); CHOS-enriched pangasius surimi gel with 0.03% Q (Q3); CHOS-enriched pangasius surimi gel with 0.04% Q (Q4); CHOS-enriched pangasius surimi gel with 0.05% Q (Q5).

The effects of Q treatment on EMC and WHC of CHOS-enriched pangasius surimi gel are presented in Figure 1. The EMC in the present study decreased progressively with increasing Q concentration, reaching the lowest value at Q4 (5.65%), which was significantly lower than that of the control (*p* < 0.05). Correspondingly, the WHC increased significantly from 85.22% in the control to a maximum of 90.66% at Q4, indicating enhanced water retention. Although a slight reduction in WHC was observed at the highest Q concentration (Q5), the value remained higher than that of the control. This inverse relationship between EMC and WHC is consistent with previous findings reporting a negative correlation between EMC and WHC in gel systems [39]. Similar trends have also been reported in other protein-based gel systems enriched with Q or polyphenol-rich extracts. Chen et al. [23] demonstrated that polyphenol incorporation enhances the WHC of myosin gels by facilitating protein unfolding during heating, exposing hydrophobic groups and promoting protein aggregation, thereby strengthening the gel network. Likewise, Buamard et al. [24] observed a reduction in EMC with increasing concentrations of Q-containing ethanolic extracts from Duea ching fruit in sardine surimi gels up to an optimal level, beyond which EMC increased, suggesting disruption of gel network integrity at excessive concentrations. In contrast, Mazumdar et al. [40] reported a decrease in WHC in pangasius gels at higher Q concentrations (0.075%) derived from onion extracts, indicating that an excessive polyphenol addition may hinder gel network formation. This phenomenon was further supported by Singh et al. [30], who showed that hyperoside (quercetin-3-O-galactoside) enhanced WHC at moderate concentrations (1–2%) through hydrogen bonding (H-bonding) between hydroxyl groups and water molecules, whereas higher concentrations interfered with the formation of an ordered gel matrix, reducing water retention. Collectively, these findings suggest that moderate Q incorporation promotes protein cross-linking and gel network formation, leading to reduced EMC and improved WHC, while an excessive addition may disrupt the gel structure and diminish WHC.

#### 2.1.6. Trichloroacetic Acid (TCA) Soluble Peptide Content, Ca^2+^-ATPase Activity, and Total Sulfhydryl Content (TSC)

Table 5 shows the effects of different levels of Q on TCA-soluble peptide content, Ca^2+^ -ATPase activity, and TSC of CHOS-enriched pangasius surimi gel. The TCA soluble peptide content decreased significantly with an increasing Q concentration (*p* < 0.05), indicating reduced protein degradation. The control sample exhibited the highest value (1.22 mg/g), while the lowest value was observed in Q4 (0.04% Q) at 1.08 mg/g. A marginal increase was noted in Q5 (1.14 mg/g), although the value remained significantly lower than the control. The decreased values indicate substantial attenuation of protein degradation, predominantly resulting from endogenous protease [41]. Covalent cross-linking between phenolic compounds and amino acids can be effectively utilized to prevent the degradation of surimi gels [42]. Q, a phenolic compound, contains multiple active phenolic hydroxyl groups and a catechol moiety on its B ring, conferring a strong capacity to interact with protein side chains [43]. In the present study, the mild oxidative conditions encountered during surimi preparation may have promoted the oxidation of the catechol structure of Q to quinone, which could subsequently react with the amino acid side chains of proteins to form more stable covalent bonds [44]. This covalent bonding might have limited the exposure of proteins to proteolytic enzymes, thereby protecting them from degradation. Conversely, Ca^2+^-ATPase activity increased significantly with Q addition up to Q4 (*p* < 0.05), suggesting better preservation of myofibrillar protein integrity. The control showed the lowest activity (1.12 μmol Pi/mg protein/min), which gradually increased through Q1, Q2, and Q3, reaching the highest value in Q4 (1.52 μmol Pi/mg protein/min). However, a decline was observed in Q5 (1.27 μmol Pi/mg protein/min), indicating that excessive Q supplementation may adversely affect enzyme activity. During surimi production, unavoidable oxidative reactions can alter the spatial structure of myosin, resulting in reduced Ca^2+^-ATPase activity [45]. A study conducted at temperatures below 48 °C, with the incorporation of 10 μmol/g Q, slightly increased the storage modulus (G′) compared with the control, indicating enhanced interactions among myosin heads and improved protein network formation [17]. Similarly, in the present investigation, Ca^2+^-ATPase activity was enhanced with the incorporation of Q at an optimized level. The TSC exhibited a significant decreasing trend with an increasing Q concentration (*p* < 0.05). The control sample had the highest sulfhydryl content (14.80 μmol/g), whereas Q4 recorded the lowest value (11.61 μmol/g). The Q5 treatment showed a slight recovery (13.13 μmol/g) but remained significantly lower than the control. Non-covalent and covalent whey protein concentrate–Q complexes, prepared with varying Q concentrations, exhibited a reduction in sulfhydryl content that may be attributed to the polyhydroxylated nature of Q [44]. Moreover, Q can form intermolecular H-bonds with proteins, and the interaction of the 5,7-hydroxyl groups on its A ring with protein sulfhydryl groups may have contributed to the observed reduction in sulfhydryl content, particularly in the non-covalent complexes [46].

#### 2.1.7. Molecular Interactions

**Figure 2:**
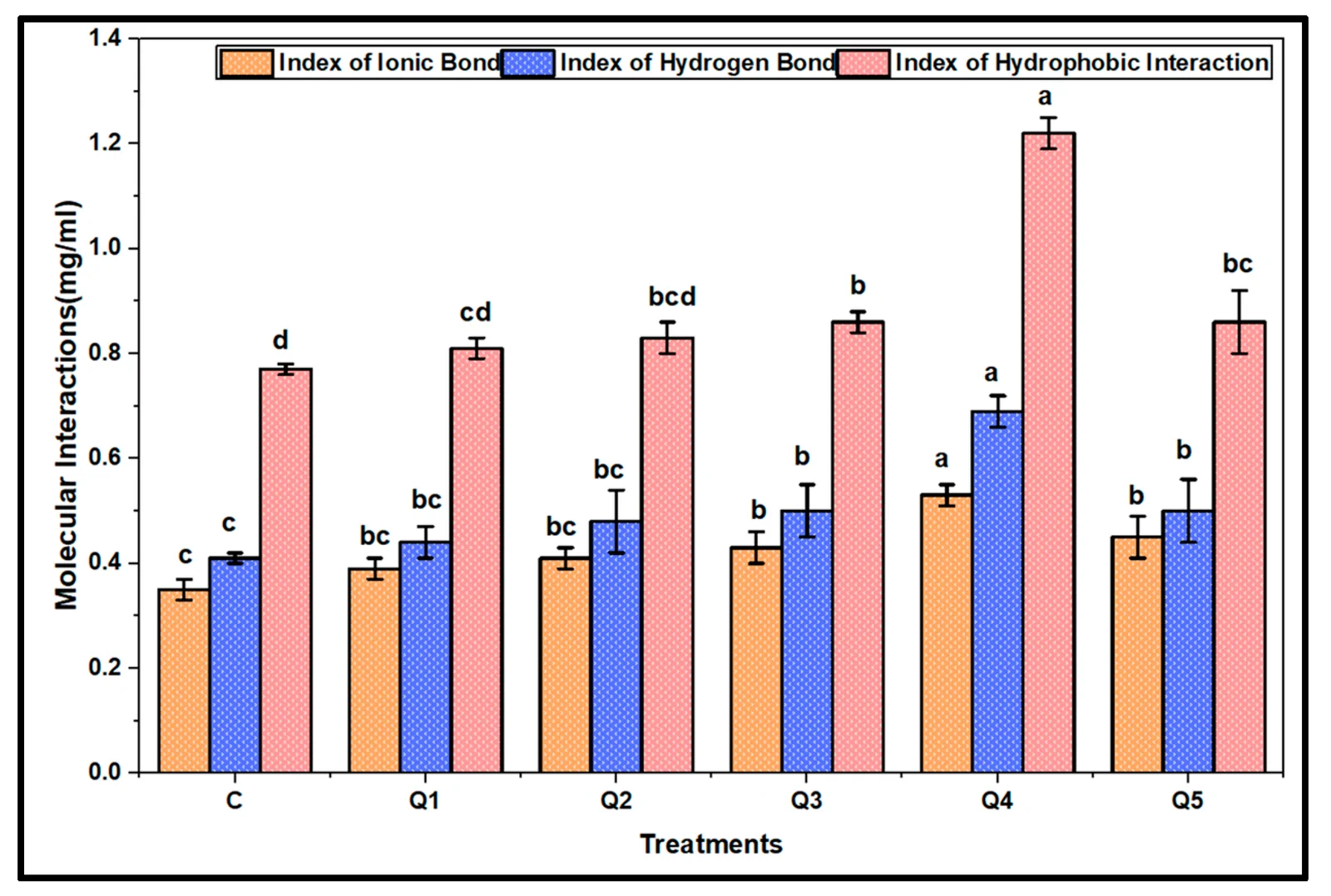
Effects of different levels of Q on the molecular interaction of CHOS-enriched pangasius surimi gel. Values are expressed as means ± standard deviations (n = 3). Different letters on the symbols indicate that the values are significantly different (*p* ˂ 0.05) between the treatments. CHOS-enriched pangasius surimi gel without Q (C); CHOS-enriched pangasius surimi gel with 0.01% Q (Q1); CHOS-enriched pangasius surimi gel with 0.02% Q (Q2); CHOS-enriched pangasius surimi gel with 0.03% Q (Q3); CHOS-enriched pangasius surimi gel with 0.04% Q (Q4); CHOS-enriched pangasius surimi gel with 0.05% Q (Q5).

The effects of Q at varying concentrations (0.01–0.05%) on the indices of ionic bonds, H- bonds, and hydrophobic interactions are presented in Figure 2. The incorporation of Q resulted in a significant enhancement of all interaction indices compared with the surimi gel control. The ionic bond index increased progressively from 0.35 mg/mL in the control to 0.53 mg/mL at Q4 (0.04%), which was significantly higher than all other treatments (*p* < 0.05). The presence of ionic bonds, but at a lower level, might be due to the salt ions that could interact with oppositely charged groups of protein molecules, influencing the ionic bonds formed between protein molecules [47]. Similarly, the H-bond index showed a concentration-dependent increase, reaching a maximum value of 0.69 mg/mL at Q4. The observed increase in H-bonding in the present study is consistent with the findings of Chen et al. [23], who reported that the incorporation of Q (4 mg/mL) into myosin (60 mg/mL) significantly enhanced the H-bond content compared with other treatments, highlighting the critical role of phenolic hydroxyl groups in flavonols in promoting H-bond formation within the myosin gel. Further, an increase in the concentration of Q results in a decrease in the H-bonds, which is attributed to the breakdown between carbon groups and amino groups in protein chains during heating [48]. Q has a distinct chemical structure featuring two benzene rings (A and B) linked by a heterocyclic pyran ring (C) with five hydroxyl groups attached at positions 3, 3′, 4′, 5, and 7 [49]. These polar groups enable extensive H-bonding with polar amino acid residues at the protein binding site (e.g., Asp, Glu, Asn, Gln, Ser, Thr, His, Lys, Arg), ensuring proper orientation and stable binding of Q. The index of hydrophobic interaction exhibited the most significant change, increasing from 0.77 mg/mL in the control to 1.22 mg/mL at Q4 in surimi gels. The predominance of hydrophobic interactions over ionic and H-bonds highlights their crucial role in surimi gel formation. By driving protein cross-linking, these interactions enhance gelation behaviour and support the establishment of a stable gel matrix [50]. Additionally, the planar aromatic rings facilitate strong hydrophobic interactions and π–π stacking with non-polar residues within hydrophobic regions of the protein (e.g., Phe, Tyr, Leu, Ile, Pro, Met, Ala) [51]. The decrease in hydrophobic interactions in Q5 could be due to the higher concentrations of Q being over-cross-linked with myofibrillar protein, which caused aggregation and denaturation of the myofibrillar protein molecules, and further folding to bury the hydrophobic amino acids in the interior [52].

#### 2.1.8. Fourier Transform Infrared (FT-IR) Spectroscopy

**Figure 3:**
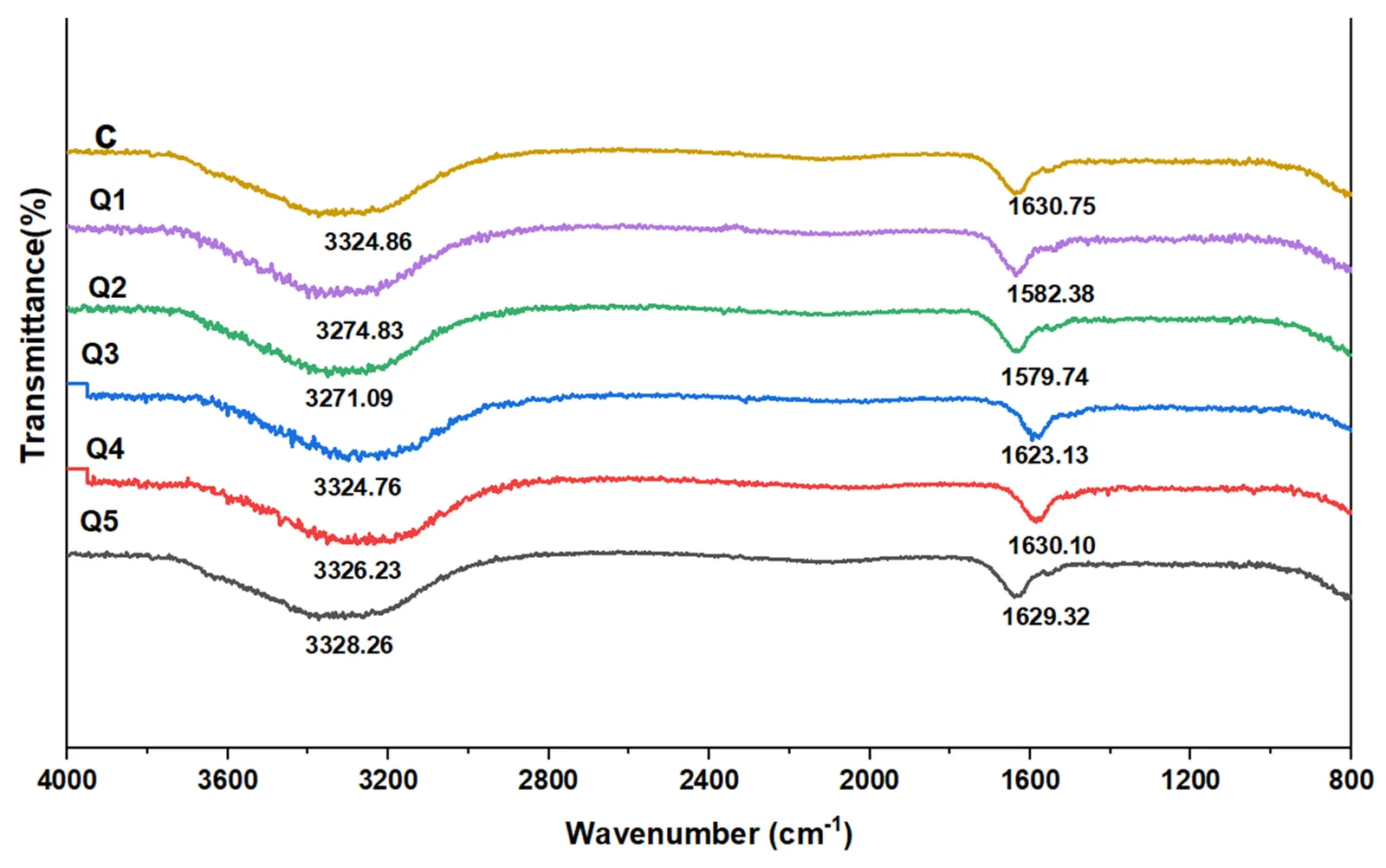
Effects of different levels of Q on the FT-IR spectra of CHOS-enriched pangasius surimi gel. CHOS-enriched pangasius surimi gel without Q (C); CHOS-enriched pangasius surimi gel with 0.01% Q (Q1); CHOS-enriched pangasius surimi gel with 0.02% Q (Q2); CHOS-enriched pangasius surimi gel with 0.03% Q (Q3); CHOS-enriched pangasius surimi gel with 0.04% Q (Q4); CHOS-enriched pangasius surimi gel with 0.05% Q (Q5).

The effects of different levels of Q on FT-IR spectra of CHOS-enriched pangasius surimi gel are presented in Figure 3. Incorporation of Q showed noticeable changes in the characteristic amide A and amide I bands compared to the control (C), indicating interactions between Q and surimi proteins. The control sample exhibited the amide A band at 3324.86 cm^−1^ and the amide I band at 1630.75 cm^−1^. In Q1 and Q2, the amide A band shifted to lower wavenumbers (3274.83 and 3271.09 cm^−1^, respectively), accompanied by shifts in the amide I region to 1582.38 and 1579.74 cm^−1^. The observed red shift in the amide A band suggests enhanced H-bonding interactions between Q and myofibrillar proteins, resulting in changes in the O-H stretching vibrations [53]. The peaks at 1582.38 and 1579.74 cm^−1^ correspond to the aromatic ring vibrations of Q molecules, confirming the incorporation of Q into the protein matrix [54]. Furthermore, the amide II region (1500–1600 cm^−1^), mainly associated with N-H vibrations, indicates possible covalent interactions between Q and myofibrillar proteins [55]. At higher Q concentrations (Q3–Q5), the amide A band shifted toward higher wavenumbers (3324.76–3328.26 cm^−1^), while the amide I band appeared around 1623.13–1630.10 cm^−1^. The blue shift, observed from 3324.76 cm^−1^ to 3326.23 cm^−1^, suggests increased non-polarity and enhanced hydrophobicity in the surimi gel system [56]. In addition, the spectral changes indicate that interactions occurred between Q and myofibrillar proteins, leading to disruption of conjugated double bonds and alteration of the external polar environment. These structural modifications promoted unfolding and expansion of the myofibrillar proteins, resulting in the gradual exposure of aromatic amino acid side chains, which may further enhance hydrophobic interactions between Q and protein molecules [57]. Additionally, recent investigations of polyphenol-protein systems corroborate these band assignments and the underlying mechanisms. Son et al. [58] observed a broad absorption band around 3270 cm^−1^, attributed to O-H stretching vibrations, in chicken breast protein–polyphenol conjugates and noted that this band indicates extensive H-bonding, the intensity of which increased with polyphenol incorporation, thereby improving the hydration and thermal stability of the protein matrix. This band position is remarkably close to the red-shifted amide A values (3274.83 and 3271.09 cm^−1^) recorded for Q1 and Q2 in the present study, confirming that the low-dose Q-induced shift reflects strengthened H-bonding rather than random spectral variations. Similarly, Zhu et al. [59] characterized the FT-IR signatures of sea bass myofibrillar protein–polyphenol complexes, assigning the amide A band centred around 3462 cm^−1^, the amide I band (C=O stretching) at 1647 cm^−1^, and the amide II band at 1544 cm^−1^, and demonstrated that polyphenol binding perturbs these characteristic frequencies, supporting the assignment of the present amide I and amide II changes to genuine protein–polyphenol complexation. Furthermore, Zhao et al. [29] reported that pepper leaf polyphenols altered porcine myofibrillar protein conformation under oxidative conditions, through the exposure of hydrophobic groups and the conversion of α-helices to β-turn structures, while molecular docking identified arginine, glutamic acid, aspartic acid, and glutamine as the predominant phenolic binding sites. These findings systematically explain the blue shift in the amide A band and the enhanced hydrophobicity observed at higher Q concentrations in the present gels, where progressive protein unfolding exposes hydrophobic residues that promote Q–protein interactions. Importantly, the observed spectral changes may also reflect interactions between Q and CHOS in the surimi gel matrix. The phenolic hydroxyl groups of Q can interact with the amino and hydroxyl groups of CHOS through H-bonding and other non-covalent interactions, thereby altering the vibrational environment of these functional groups. Such Q–CHOS interactions may contribute to the shifts and intensity changes observed in the amide A and amide-related regions, supporting the formation of a Q–CHOS-protein interaction network within the surimi gel matrix.

#### 2.1.9. Thermal Stability Properties

The thermal stability of CHOS-enriched pangasius surimi sol, as influenced by different levels of Q (Q1-Q5), is presented in Table 6 through three endothermic transition peaks (Peak-I, Peak-II, and Peak-III). This showed noticeable variations in peak temperatures and enthalpy values compared to the control. The control sample exhibited endothermic peak temperatures at 51.70 and 57.10 °C corresponding to the denaturation of the myosin (head and tail), with enthalpies of −23.40 and −7.14 J/g, respectively [60]. The third peak is due to actin at 93.10 °C, with an enthalpy value of −548.44 J/g. The addition of salt to surimi may lead to a higher actin denaturation temperature [60]. The disappearance of the myosin peaks in Q1 was attributed to the aggregation of myosin head and tail caused by phenolic compounds and further shifted to a higher temperature, indicating that the thermal stability of myofibrillar protein was improved [61]. In Q2 and Q3, the peak temperatures shifted towards higher temperatures compared to the control. Q2 exhibited peaks at 56.20, 78.50, and 91.90 °C, while Q3 showed peaks at 65.40, 72.00, and 96.20 °C. Q4 demonstrated a peak at 67.70 °C and a major peak at 96.00 °C with a markedly high enthalpy value (−2075.01 J/g), indicating the formation of a highly stable protein network. According to the findings of Wu et al. [62], Q develops a non-covalent protein network through H-bonds and hydrophobic interactions, resulting in improved conformational stability. Consequently, the Q–protein complex exhibits a higher thermal dissociation energy barrier and greater resistance to heat-induced unfolding. However, in Q5, the peak temperatures decreased to 51.60, 57.70, and 92.00 °C with substantially altered enthalpy values, suggesting that an excessive Q concentration may have destabilized the protein structure or caused aggregation, thereby reducing thermal stability. ΔH reflects the breaking of H-bonds and the gradual unfolding of the protein network during the denaturation process [63]. The lowest enthalpy was observed for Q5 for myosin peaks. The decrease in ∆H, reflecting lower energy absorption during gelation, indicates disruption of the gel network as the concentration of Q increases [15]. Moreover, these thermal transitions are corroborated by recent findings on polyphenol–myofibrillar protein systems. Su et al. [55] demonstrated that covalent conjugation with Q improved the thermal stability of rabbit myofibrillar proteins, as the solubility of heat-treated proteins increased from 18.34% to 80.53% with increasing Q levels, whereas an excessive dose (100 μmol/g protein) reduced solubility to 68.33% because of excessive cross-linking between the myosin head and tail. This mechanism is analogous to the disappearance and upward shifting of the myosin peaks observed in Q1-Q4 and their subsequent destabilization in Q5 in the present study. Likewise, Chen et al. [64] reported that increasing concentrations of green tea polyphenols in protein gels progressively reduced the onset and denaturation temperatures, together with the denaturation enthalpy (ΔH decreased from 7.13 to 6.45 J/g at pH 5), which is consistent with the reduced ΔH and altered peak positions observed at higher Q concentrations in the present study. Moreover, Zhu et al. [65] demonstrated that gelatin–tea polyphenol complexes stabilized surimi myofibrillar proteins through H-bonding between amino groups and phenolic hydroxyl groups, thereby enhancing protein structural stability against heat- and ice-induced damage. Collectively, these studies support the view that moderate Q concentrations (Q1-Q4) strengthen the Q–protein network and increase its thermal dissociation barrier, whereas Q5 exceeds the concentration threshold at which beneficial cross-linking is replaced by aggregation-induced destabilization of the gel matrix (Figure S1).

#### 2.1.10. Protein Patterns

**Figure 4:**
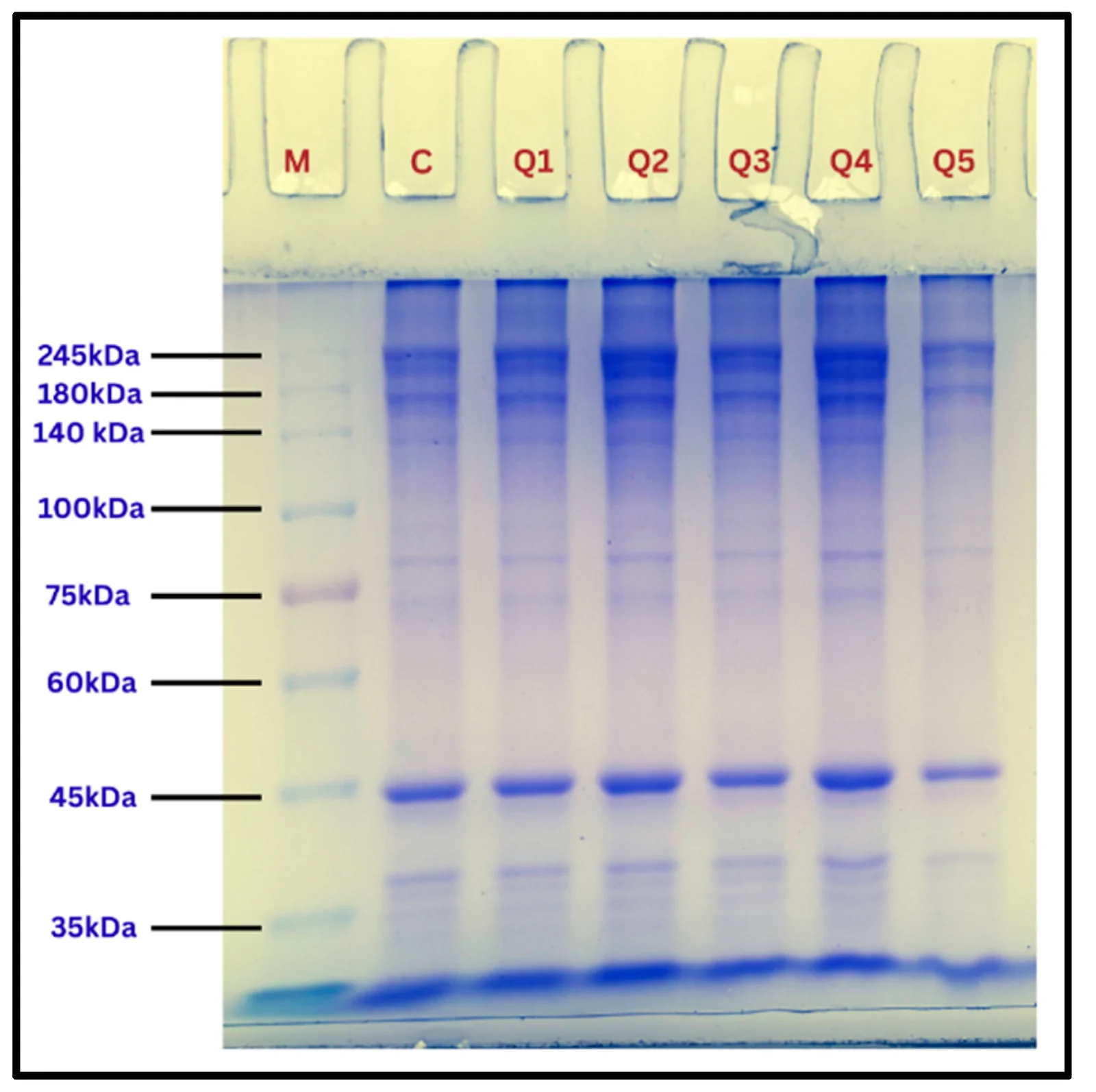
Effects of different levels of Q on the protein patterns of CHOS-enriched pangasius surimi gel. Protein markers (M); CHOS-enriched pangasius surimi gel without Q (C); CHOS-enriched pangasius surimi gel with 0.01% Q (Q1); CHOS-enriched pangasius surimi gel with 0.02% Q (Q2); CHOS-enriched pangasius surimi gel with 0.03% Q (Q3); CHOS-enriched pangasius surimi gel with 0.04% Q (Q4); CHOS-enriched pangasius surimi gel with 0.05% Q (Q5).

The SDS-PAGE profiles (Figure 4) illustrate the effects of different concentrations of Q on the protein patterns of CHOS-enriched pangasius surimi gel. The control sample exhibited distinct bands for myosin heavy chain (MHC, ~200 kDa) and actin (~45 kDa). With an increasing Q concentration, the intensity of the MHC band gradually decreased, reaching the lowest level in Q4 (0.04% Q), while the accumulation of high-molecular-weight material near the top of the gel became more pronounced. The diminished MHC band intensity in Q-treated samples suggests that Q promoted the formation of high-molecular-weight protein aggregates (HMMA) through covalent cross-linking, thereby reducing the migration of MHC during electrophoresis. Similar findings have been reported for Q-myofibrillar protein conjugates, where Q preferentially interacts with MHC, promoting protein polymerization and the formation of HMMAs. These aggregates are generated through both disulfide bond formation and non-disulfide covalent interactions involving C-N and/or C-S linkages between oxidized Q quinones and nucleophilic amino acid residues [55]. In contrast, the relatively unchanged intensity of the actin (~45 kDa) band across treatments suggests that actin was less susceptible to direct Q modification and contributed minimally to covalent aggregate formation. Zainudin et al. [66] demonstrated that a cross-linked MHC band was not detectable in control samples lacking the polyphenol 4-methylcatechol, where only reversible disulfide cross-links were formed. In contrast, phenol-mediated protein cross-linking under oxidative conditions generated covalent protein–polyphenol bonds that altered the textural and functional properties of myofibrillar proteins. Likewise, Somjid et al. [67] reported that the MHC bands of all gelled frigate mackerel surimi samples were thinner than those of the corresponding non-gelled samples. This reduction in band intensity was attributed to protein polymerization and cross-linking during thermal gelation, while a portion of the aggregates remained trapped in the stacking gel, even under reducing conditions because of non-disulfide hydrophobic interactions. This pattern closely resembles the progressive loss of the MHC band and the accumulation of HMMAs at the top of the gel observed in the present Q-treated samples. Systematically, Wu et al. [9] explained that the o-quinones generated during the oxidation of phenolic compounds can react with protein-bound nucleophiles and subsequently conjugate with additional protein molecules, thereby promoting protein cross-linking. In the present system, oxidized Q-derived quinones may therefore function as molecular bridges between adjacent myosin molecules through C-N and/or C-S linkages. In contrast, the limited participation of actin in covalent aggregate formation may reflect its lower reactivity toward quinone adduction, which is consistent with the essentially unchanged actin band intensity observed across treatments.

#### 2.1.11. Scanning Electron Microscopy (SEM)

**Figure 5:**
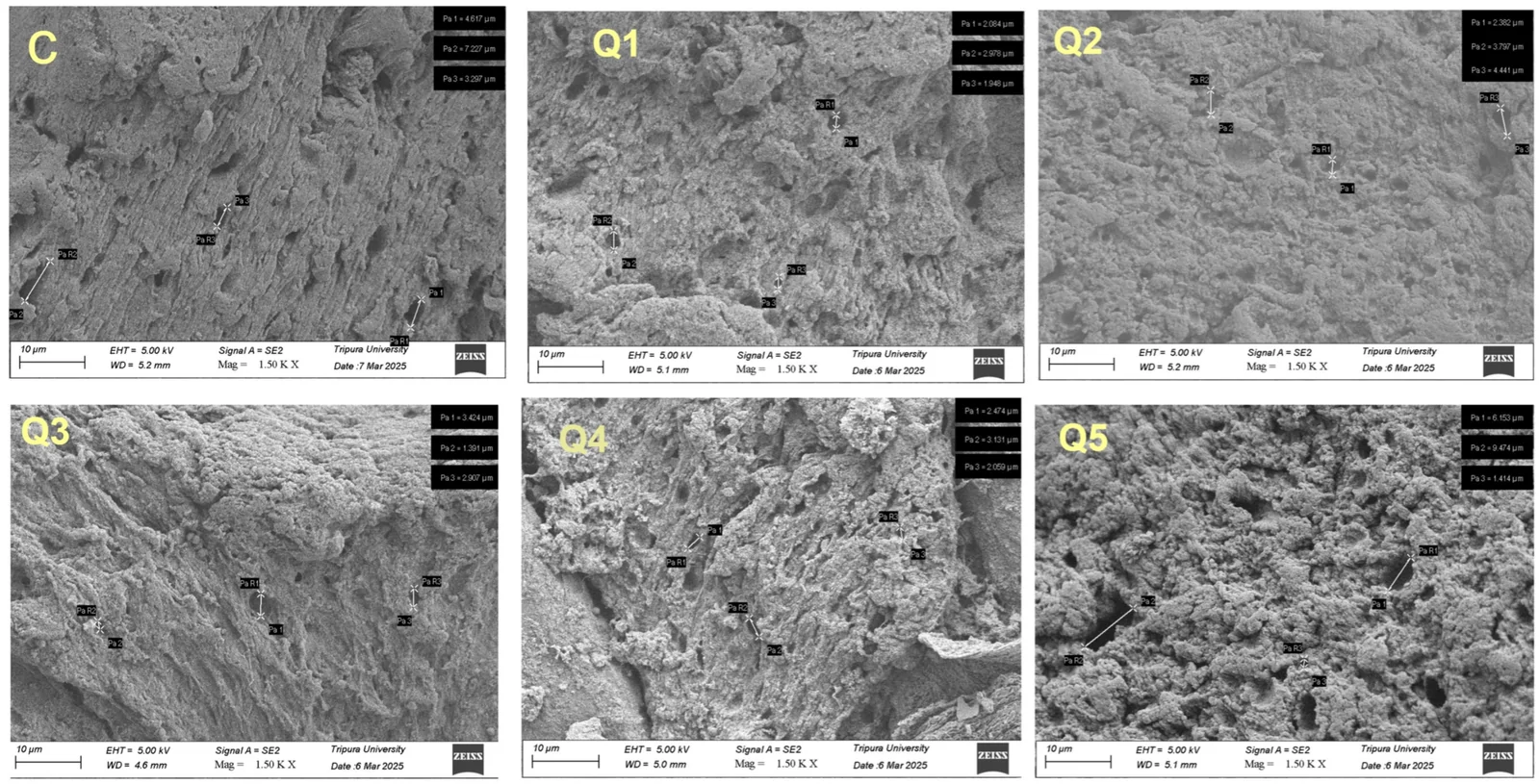
Effects of different levels of Q on SEM of CHOS-enriched pangasius surimi gel. CHOS-enriched pangasius surimi gel without Q (C); CHOS-enriched pangasius surimi gel with 0.01% Q (Q1); CHOS-enriched pangasius surimi gel with 0.02% Q (Q2); CHOS-enriched pangasius surimi gel with 0.03% Q (Q3); CHOS-enriched pangasius surimi gel with 0.04% Q (Q4); CHOS-enriched pangasius surimi gel with 0.05% Q (Q5).

The SEM of CHOS-enriched pangasius surimi gel treated with different levels of Q is presented in Figure 5. All samples were observed at a magnification of 1500× with a 10 µm scale bar. The control sample exhibited a relatively coarse and heterogeneous network with irregular pores and loosely arranged protein strands. The incorporation of Q gradually enhanced the gel matrix’s compactness by promoting protein aggregation and intermolecular interactions. Among the treatments, Q4 (0.04% Q) exhibited the most compact and homogeneous network with fewer, smaller voids, whereas the Q5 treatment showed a rougher, more granular structure with larger pores. This phenomenon may be attributed to rapid, extensive protein cross-linking, resulting in a densely coagulated protein network. These findings are in agreement with previous studies on polyphenol-enriched surimi gels. Buamard et al. [24] reported that the incorporation of *Duea ching* (*Ficus botryocarpa* Miq.) extract, rich in polyphenolic compounds such as naringenin-7-*O*-glucoside, quercetin 3-galactoside (hyperoside), and rutin, promoted protein aggregation and resulted in a more compact gel network. Similarly, Priyadarshini et al. [68] observed a comparable microstructural pattern in tilapia surimi supplemented with an optimal level of 60% ethanolic plant extract containing bioactive phenolics, including α-amino diphenylacetic acid, 3-O-methyl rimiterol, dihydromyricetin, and dihydroquercetin, which enhanced the structural integrity of the protein matrix. The concentration-dependent microstructural transition observed in the present investigation is corroborated by recent SEM studies of polyphenol-modified protein gels. Lv et al. [33] reported that polyphenol-treated *Penaeus vannamei* myosin gels exhibited smoother gel surfaces than the control under SEM, accompanied by increased WHC, indicating a better-organized gel matrix that is consistent with the compact, void-poor network recorded for Q4 in the present study. Likewise, Cheng et al. [69] demonstrated that Q modification of oxidized myofibrillar protein produced a dense, uniform microstructure that sustained gel-forming capacity, confirming that appropriate polyphenol levels refine, rather than disrupt, the protein network. Conversely, Tian et al. [34] observed that an excessive level of EGCG (0.03%) produced a loose protein network with moisture loss in silver carp surimi gels, whereas lower doses (0.01–0.02%) preserved the structural integrity pattern directly analogous to the rougher, granular structure with larger pores seen in Q5, where the balance shifts from orderly aggregation to dense coagulation. Together, these studies support the interpretation that 0.04% Q induces constructive protein–protein cross-linking that densifies the gel matrix, while 0.05% triggers excessive coagulation that degrades the microstructure.

#### 2.1.12. Antioxidant Activities

**Figure 6:**
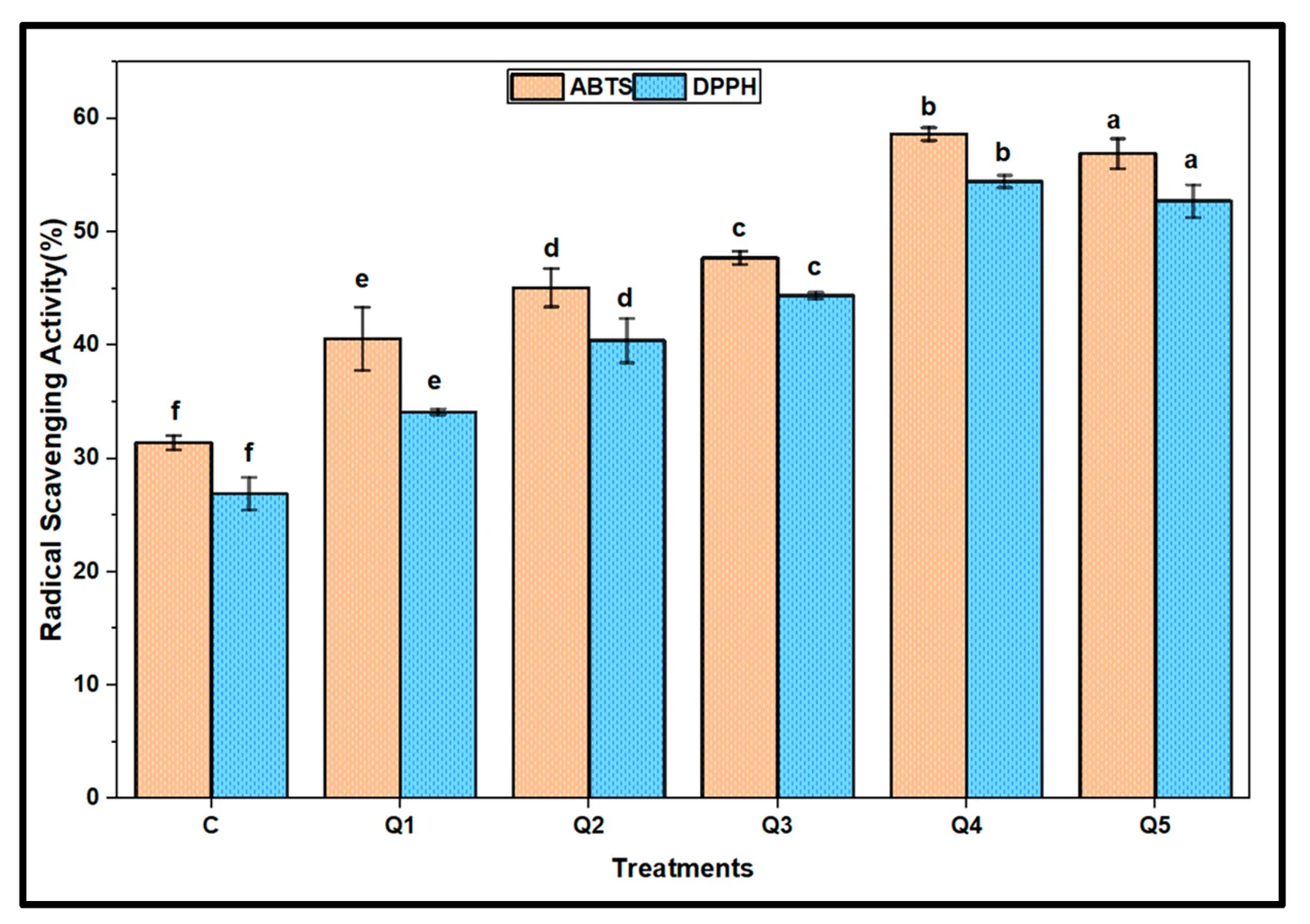
Effects of different levels of Q on the antioxidant activities of CHOS-enriched pangasius surimi gel. Values are expressed as means ± standard deviations (n = 3). Different letters on the symbols indicate that the values are significantly different (*p* ˂ 0.05) between the treatments. CHOS-enriched pangasius surimi gel without Q (C); CHOS-enriched pangasius surimi gel with 0.01% Q (Q1); CHOS-enriched pangasius surimi gel with 0.02% Q (Q2); CHOS-enriched pangasius surimi gel with 0.03% Q (Q3); CHOS-enriched pangasius surimi gel with 0.04% Q (Q4); CHOS-enriched pangasius surimi gel with 0.05% Q (Q5).

The antioxidant activities of CHOS-enriched samples treated with different levels of Q were evaluated using ABTS and DPPH radical scavenging assays, and the results are presented in Figure 6. The Q addition significantly enhanced both ABTS and DPPH radical scavenging activities compared with the control (*p* < 0.05). ABTS scavenging activity increased from 31.39% in the control to 58.62% at Q4, showing a distinct concentration-dependent improvement. A slight but non-significant decrease was observed at Q5, although values remained significantly higher than those of lower concentrations. Similarly, DPPH scavenging activity increased markedly with increasing Q concentration, rising from 26.87% in the control to a maximum of 54.44% at Q4. No significant difference was observed between Q4 and Q5, indicating that antioxidant activity reached a plateau at higher Q levels. The observed differences reflect distinct radical scavenging mechanisms, whereby DPPH scavenging is dominated by hydrogen atom transfer reactions, while ABTS and hydroxyl radical scavenging depend largely on electron transfer and metal ion-associated redox mechanisms [70]. The antioxidative activity of Q is mainly contributed by the hydroxyl groups in the C-ring and a catechol group in the B ring [71]. The increase in antioxidant capacity may be because the complex enhanced the dispersity of Q molecules in aqueous solutions, making them more likely to interact with free radicals produced in the surrounding aqueous phase [72]. The increasing Q concentration led to a corresponding increase in DPPH activity, indicating that Q enhances antioxidant capacity. In the present study, the result is consistent with the work of Yang et al. [73], who demonstrated that non-covalent binding between *sacha inchi* protein and Q confers greater radical scavenging ability than the protein alone. The pronounced enhancement (31.39–56.89%) in ABTS radical scavenging activity is primarily attributed to the availability of abundant hydroxyl groups in Q, which markedly strengthen its free radical neutralizing capacity. Similar findings have been reported for Q–coconut protein complexes, which exhibited higher antioxidant activity than free Q, likely because the protein matrix improves Q solubility and facilitates its interaction with free radicals [9,74]. In addition, Rani et al. [75] observed a concentration-dependent increase in ABTS scavenging activity following the incorporation of Q (1–3%) into soy protein isolate films, further supporting the role of Q concentration in enhancing antioxidant capacity. Overall, these results demonstrate that Q effectively enhances antioxidant capacity, with 0.04–0.05% identified as the optimal concentration range for maximizing radical scavenging activity. These concentration-dependent trends are broadly consistent with recent polyphenol-fortified fish gel studies. Hong et al. [76] reported that black tea powder added to silver carp fish balls significantly increased antioxidant activity in a dose-dependent manner, with the strongest effects at 0.3%, where the DPPH, ABTS, and hydroxyl radical scavenging rates reached 57.93%, 89.24%, and 50.64%, respectively; effects that are attributed to the phenolic compounds of black tea powder. Likewise, Lv et al. [33] showed that cold atmospheric plasma treatment activated the phenolic hydroxyl groups of tea polyphenols in squid surimi, enhancing ABTS scavenging activity by 38.6% and DPPH scavenging by 8.41% while preserving protein integrity and gel structure. Notably, in omega-3-enriched fish gels, Q-supplemented gels exhibited the highest antioxidant capacity when assayed by the ferric-reducing/antioxidant power method, whereas DPPH values were not elevated and no prooxidative effect was evident in vivo, with plasma malondialdehyde remaining unchanged [35]. This assay-dependent response underscores the present observation that ABTS and DPPH scavenging proceed through distinct mechanisms, and the plateau in DPPH activity between Q4 and Q5 is consistent with the saturation of the phenolic hydroxyl groups that drive radical neutralization, which is in line with the mechanistic basis invoked for Q’s antioxidative action in this study.

#### 2.1.13. Antimicrobial Activity

**Figure 7:**
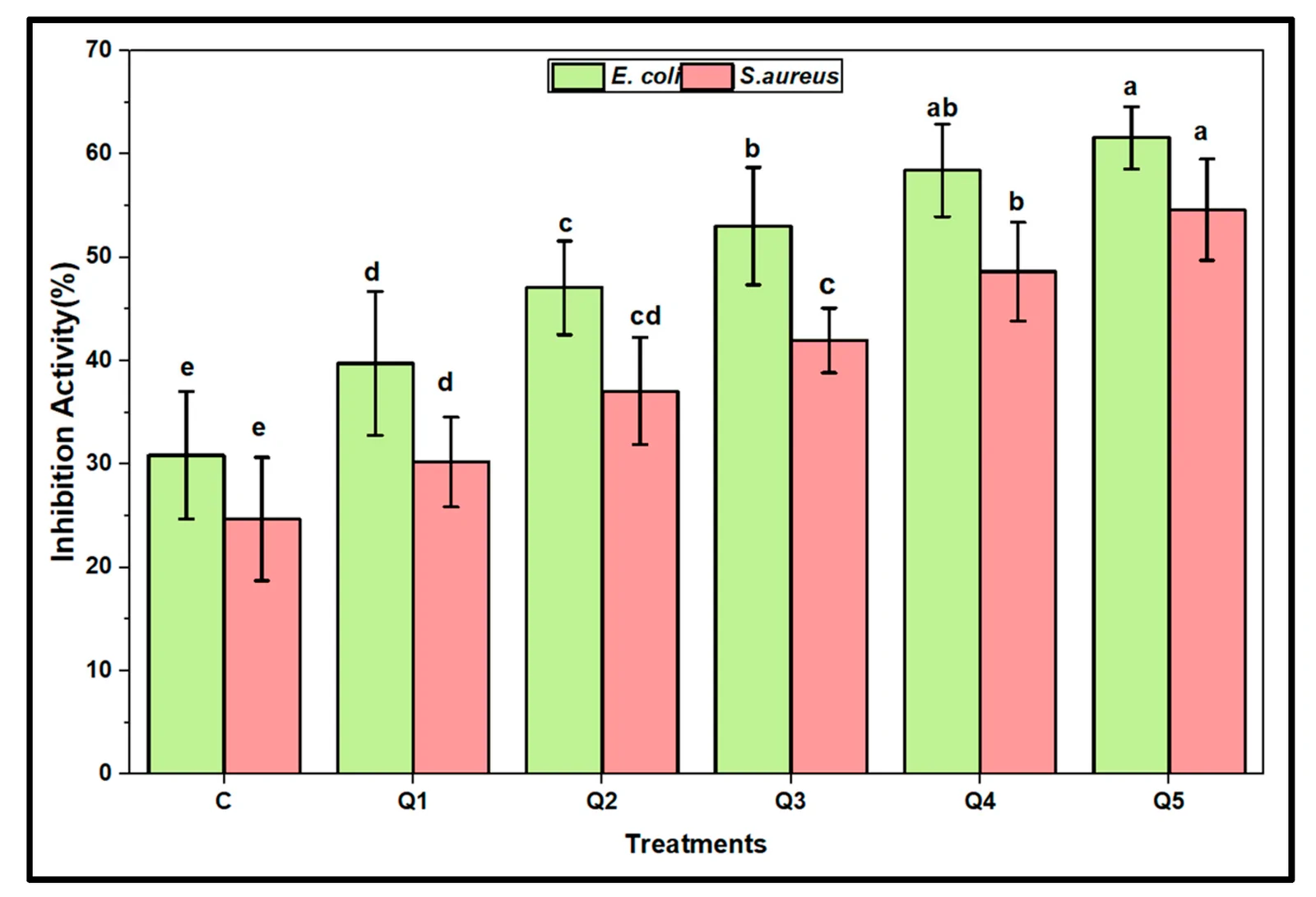
Effects of different levels of Q on the antimicrobial activities of CHOS-enriched pangasius surimi gel. Values are expressed as means ± standard deviations (n = 3). Different letters on the symbols indicate that the values are significantly different (*p* ˂ 0.05) between the treatments. CHOS- enriched pangasius surimi gel without Q (C); CHOS-enriched pangasius surimi gel with 0.01% Q (Q1); CHOS-enriched pangasius surimi gel with 0.02% Q (Q2); CHOS-enriched pangasius surimi gel with 0.03% Q (Q3); CHOS-enriched pangasius surimi gel with 0.04% Q (Q4); CHOS-enriched pangasius surimi gel with 0.05% Q (Q5).

The antibacterial activity of Q-incorporated surimi gel against *Escherichia coli* (Gram-negative) and *Staphylococcus aureus* (Gram-positive) is presented in Figure 7. The Q-enriched surimi gel significantly enhanced the inhibition rates against both *E. coli* and *S. aureus* compared with the control. For *E. coli*, the inhibition rate increased from 30.87% in the control to 61.59% at Q5 (0.05%), showing a clear concentration-dependent pattern. Significant improvements were observed from Q2 onward (*p* < 0.05). Correspondingly, the inhibition of *S. aureus* increased with increasing Q concentration, rising from 24.69% in the control to 54.61% at Q5. Although lower concentrations showed moderate antibacterial effects, the highest inhibition was achieved at 0.05% Q. The antibacterial effects observed in the present study are consistent with previous reports describing the broad-spectrum antimicrobial activity of Q. The significant inhibition of both *E. coli* and *S. aureus* with increasing Q concentration can be attributed to its ability to disrupt bacterial membranes, interfere with nucleic acid and protein synthesis, suppress virulence factor expression, and inhibit biofilm formation [77]. In line with the present study findings, Yuan et al. [78] demonstrated that the antibacterial activity of flavonoids against Gram-positive bacteria is strongly associated with lipophilicity, with the bacterial cell membrane serving as the primary target through phospholipid bilayer disruption and inhibition of respiratory or ATP synthesis pathways. Furthermore, the pronounced inhibitory effect observed in this study may also be explained by Q’s capacity to alter membrane structure, fluidity, and cell size, as reported by Veiko et al. [79], thereby enhancing membrane permeability and compromising bacterial integrity. Moreover, although Q does not necessarily reduce bacterial viability, its ability to inhibit coagulase secretion and attenuate *S. aureus* virulence [80] may contribute to the elevated inhibition rates observed. At the molecular level, Q localization within hydrophobic membrane regions can induce raft-like domain formation, while its interaction at polar interfaces increases membrane fluidity [81], providing a mechanistic basis for the concentration-dependent antibacterial activity detected in this study. The antimicrobial activity of Q against *E. coli* is likely due to its ability to disrupt bacterial cell wall integrity and interfere with acid trehalase (ATH), inhibiting intracellular proteins [82]. These concentration-dependent inhibitory trends are reinforced by recent quantitative assessments of Q’s antimicrobial potency. Karpiński et al. [83] determined the minimum inhibitory concentration (MIC) of Q against a panel of pathogens, reporting moderate activity against Gram-positive bacteria (MIC 125–500 μg/mL) but much weaker activity against Gram-negative bacteria (mean MIC > 1000 μg/mL), and proposed an interpretive scale for plant-derived compounds whereby strong, moderate, and weak activity correspond to MICs of <100, 100–250, and 251–1000 μg/mL, respectively. In line with this membrane-targeted mode of action, Mahmud et al. [84] reported that Q damaged the cell walls and membranes of both *E. coli* and *S. aureus* by enhancing the production of alkaline phosphatase and β-galactosidase, an effect that increased exponentially with Q concentration, while *S. aureus* biofilm formation was inhibited by more than 70% at a dose of 20 μg/mL. Similarly, comparative testing of Q and related polyphenols showed stronger inhibition of Gram-positive than of Gram-negative bacteria, although a pronounced bacteriostatic effect against *E. coli* could still be achieved, attributed to inhibition of D-alanine ligase and prevention of bacterial growth [85]. Collectively, these findings substantiate the dose-dependent, membrane-disruptive mechanism underlying the rising inhibition rates observed in the present study and support 0.05% Q as an effective antimicrobial level in CHOS-enriched pangasius surimi gel.

## 3. Conclusions

This study demonstrates that the direct incorporation of Q into pangasius surimi gel, enriched with CHOS derived from snow crab shells, serves as a viable and efficient alternative to the complex CHOS-phenolic conjugation process. The addition of Q enhances the gel’s physicochemical, textural, structural, thermal, antioxidant, and antimicrobial properties, with effects that vary with concentration. At an optimal concentration of 0.04% Q (Q4), the gel achieves maximum strength, improved texture, superior water retention, enhanced protein stability, stronger molecular interactions, a more compact microstructure, and increased thermal stability. These improvements are primarily attributed to enhanced H- bonding, hydrophobic interactions, and protein cross-linking between Q and myofibrillar proteins. Additionally, Q significantly enhances the antioxidant and antimicrobial activities of the surimi gel. However, higher concentrations may result in reduced whiteness and a slight decline in gel quality due to excessive protein aggregation. Thus, fortifying CHOS-enriched pangasius surimi with 0.04% Q offers a straightforward, cost-effective, and scalable approach to producing functional surimi products with improved quality, thereby enhancing value addition in freshwater surimi processing. Future research should therefore focus on sensory evaluation and consumer acceptability studies, as well as long-term storage trials, to confirm that the quality improvements and functional benefits are retained under real commercial distribution conditions. In addition, in vivo studies are needed to assess the bioavailability and safety of Q-fortified surimi gels, along with pilot-scale processing trials and molecular docking analyses that could further elucidate the interaction mechanisms and support industrial-scale adoption of this fortification strategy.

## 4. Materials and Methods

### 4.1. Schematic Overview of the Experimental Programme

**Figure 8:**
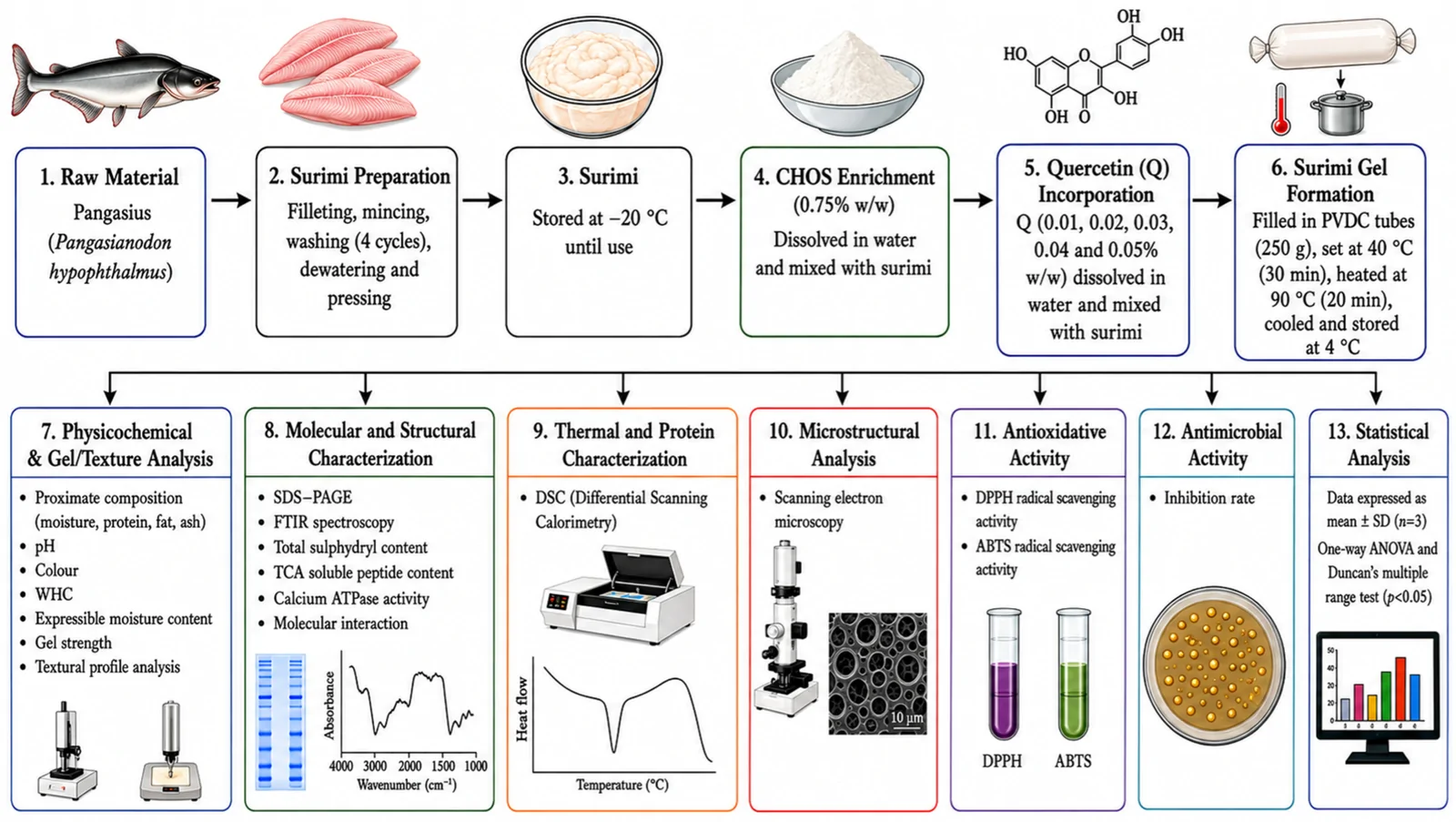
Schematic overview of the experimental programme.

Figure 8 presents a schematic overview of the complete experimental programme, encompassing raw material preparation, pangasius surimi production, CHOS enrichment, Q incorporation, gel formation, quality characterization, structural and bio-functional analyses, and statistical evaluation. The flow diagram provides a sequential representation of the experimental procedures followed to evaluate the effects of different Q concentrations on the physicochemical, gel, textural, structural, thermal, antioxidant, and antimicrobial properties of CHOS-enriched pangasius surimi gel. All chemicals and reagents used in this work were of analytical grade and procured from certified retailers. The experimental work adhered to good laboratory practices as prescribed by the Department of Fish Processing Technology and Engineering, College of Fisheries, Central Agricultural University (Imphal), Lembucherra, Agartala, Tripura, India.

### 4.2. Materials

Pangasius fish (*Pangasianodon hypophthalmus*), with a length of 59.20 ± 3.03 cm and weighing 2.32 ± 0.39 kg, were bought from Gol Bazar fish market, Agartala, India, and were carried to the fish processing laboratory, College of Fisheries, Lembucherra, Agartala, India, in ice boxes with a finely crushed ice medium. Qingdao Reach International Inc., Qingdao, China, supplied commercial CHOS powder (degree of deacetylation: 90%; Molecular weight: 3 kDa), obtained from snow crab shell, while AKI Organic™, Terrebonne, QC, Canada, provided Q (≥98% purity), a natural extract derived from *Sophora japonica* flowers.

### 4.3. Methods

#### 4.3.1. Preparation of Pangasius Surimi

The pangasius surimi was produced according to the protocols delineated by Priyadarshini et al. [86]. The chilled fish were headed, eviscerated, filleted, washed, and the fillets were processed using a mechanical deboner (Zhengzhou Taizy Machinery Co., Ltd., Zhengzhou, China) with a 3 mm perforation plate, maintaining the mince temperature at 10 °C. The minced meat was suspended in a cold (5 °C) alkaline–salt solution (0.15% NaCl and 0.2% NaHCO_3_) at a mince-to-solution ratio of 1:4 (*w*/*v*). The mixture was gently stirred for 15 min and permitted to settle for 5 min. The rinsed mince was further strained through a nylon mesh. The washing process was performed twice more. A cooled 0.5% sodium chloride solution was selected for the final rinse. The washed mince was subjected to stirring in a basket centrifuge for 10 min to eliminate any excess water. The prepared surimi was packaged in LDPE material and subsequently placed on ice for future use.

#### 4.3.2. Formulation of CHOS-Enriched Pangasius Surimi with Different Levels of Q

The surimi was properly mixed with 25 g/kg NaCl and then blended with 7.5 g/kg CHOS, as optimized in the previous study [13], to prepare the foundation material. From the foundation material, appropriate amounts of surimi paste were incorporated with varied concentrations of Q at 0.1, 0.2, 0.3, 0.4, and 0.5 g/kg, designated as Q1, Q2, Q3, Q4, and Q5, respectively. The surimi without Q was marked as the control (C). The moisture content of the final surimi paste was brought to 80%. After 2 min of chopping at a temperature below 8 °C, the paste was stuffed into a 2.5 cm polyvinylidene chloride (PVDC) casing and tied at both ends. In order to prepare the gel, the paste was first incubated at 40 °C for 30 min and subsequently heated at 90 °C for 20 min [87]. The gel was instantaneously chilled in ice-cold water for 1 h and then stored at 4 °C for 18 h, prior to analysis.

### 4.4. Quality Evaluation of CHOS-Enriched Pangasius Surimi with Different Levels of Q

#### 4.4.1. Proximate Composition and pH

The proximate composition of surimi gel, including moisture, crude protein, fat, and ash, was determined following the methods of analysis of the Association of Official Analytical Chemists International (AOAC) [88], and all data were reported on a wet weight basis. The pH of surimi gel was measured using the method outlined by Gao et al. [89]. A 5 g gel sample was mixed with 45 mL of deionized water and then homogenized with a Remi Lab Stirrer (Model RQ-127D, REMI ELECTROTECHNIK Ltd., Vasai, India) at 8000 rpm for 1 min. A pH meter (HI 5522, Hanna Instruments Inc., Woonsocket, RI, USA) was used to measure the pH.

#### 4.4.2. Gel Properties

A texture analyzer (TA, XT plus, Stable Micro Systems, Surrey, UK) was used to assess the BF and DF of surimi gels, as suggested by Vate and Benjakul [90]. Cylindrical gels with a diameter of 2.5 cm and a height of 3 cm were sectioned and equilibrated to ambient temperature for 30 min prior to investigation. A spherical plunger (type: P/5S; diameter: 5 mm; material: stainless steel) was inserted perpendicularly into the sliced surface of a gel sample at a constant speed of 1.1 mm/s. The penetration force (BF) and distance (DF) of the plunger into the gel were determined.

#### 4.4.3. Texture Profile Analysis (TPA)

Cylindrical gel samples (2.5 cm in diameter and 3 cm in height) were tested using a texture profile analyzer (TA, XT plus Stable Micro System, Surrey, UK) equipped with the SMS P/75 probe. The textural parameters, such as hardness, cohesiveness, springiness, chewiness, and gumminess, were measured by applying force–time curves. The springiness was calculated as the ratio of the time difference during the second compression to that during the first compression, and the chewiness was calculated as hardness × cohesiveness × springiness.

#### 4.4.4. Instrumental Colour Attributes and Whiteness

The whiteness of gel samples was determined utilizing a colorimeter (Colourflex EZ, Hunter Associates Laboratory, Inc., Reston, VA, USA). The values of L*, a*, and b* were computed, and subsequently, the whiteness was assessed using the equation proposed by Benjakul et al. [91]:

Whiteness = 100 − [ (100 − L*) + a*^2^ + b*^2^ ]^1/2^

where L* is the lightness, a* is the redness/greenness, and b* is the yellowness/blueness.

#### 4.4.5. Expressible Moisture Content (EMC) and Water-Holding Capacity (WHC)

The EMC and WHC of gels were evaluated according to the methodologies established by Chanarat et al. [92] and Kong et al. [93], respectively. The detailed procedure is outlined in the Supplemental Materials.

#### 4.4.6. TCA Soluble Peptide Content, Ca^2+^-ATPase Activity, and Total Sulfhydryl Content (TSC)

The TCA-soluble peptide content, Ca^2+^-ATPase activity, and TSC of the gels were analyzed using the methodologies outlined by Buamard et al. [94], Endoo and Yongsawatdigul [95], and Lin et al. [96], respectively. The elaborate procedure is outlined in the Supplemental Materials.

#### 4.4.7. Molecular Interaction

The molecular interaction of surimi gel samples was assessed using the methodology established by Liu et al. [97]. The comprehensive procedure is outlined in the Supplemental Materials.

#### 4.4.8. Fourier Transform Infrared (FT-IR) Spectroscopy

The FT-IR spectra of the gel samples were recorded using a Nicolet iS5 FT-IR spectrometer (ThermoScientific, Waltham, MA, USA) equipped with a micro-attenuated total reflectance (ATR) accessory. Surimi gel (1 mg) was blended with potassium bromide (100 mg). Then, the mixture was ground to a homogeneous mass and pressed into a diaphanous sheet using a hydraulic machine at a pressure of (5–10) × 10^7^ Pa. The spectra were acquired in the 4000–400 cm^−1^ region with a resolution of 4 cm^−1^ and averaged over 32 scans. The FT-IR sample spectra were processed and fitted by the OMNIC 9 filmware version 2.0.

#### 4.4.9. Thermal Stability Properties

The denaturation temperatures of myofibrillar proteins in surimi sol samples were determined by a Chip-DSC 10 calorimeter (Linseis, Selb, Germany). A 30 mg surimi sol sample in a hermetically sealed aluminum pan underwent DSC analysis from 20 °C to 120 °C at a scanning rate of 10 °C/min. The initiation and peak transition temperatures were recorded, and the change in enthalpy (ΔH) was determined by computing the area beneath the DSC transition curve utilizing Linseis software.

#### 4.4.10. Protein Patterns

The protein patterns of the surimi gel were visualized by SDS-PAGE, as described by Laemmli [98]. The stacking gel (4%) and separating gel (10%) were applied for the analysis. The comprehensive procedure is outlined in the Supplemental Materials.

#### 4.4.11. Scanning Electron Microscopy (SEM)

The microstructure of the surimi gels was investigated using SEM following the methodology of Chen et al. [99]. The comprehensive procedure is outlined in the Supplemental Materials. The analytical conditions are set as per the procedure of Chen et al. [100]. The dried samples were mounted onto aluminum SEM stubs using conductive carbon tape and sputter-coated with gold to render the sample surface electrically conductive. The sputter coating was performed at an accelerating voltage of 1.0 kV, sputtering current of 20 mA, sputtering time of 60 s, working distance of 40 mm, and sputtering working pressure of approximately 8 Pa. The gold-coated samples were subsequently examined using a field-emission SEM. SEM imaging was performed at an accelerating voltage of 15 kV, an emission current of 32.5 µA, a working distance of 6 mm, and an observation chamber pressure of approximately 1 × 10^−4^ Pa. Images were acquired using the secondary electron detector at a magnification of 1500×, with a 10 µm scale bar. The same instrumental and imaging conditions were maintained for all samples to ensure a reliable comparison of their microstructural characteristics.

#### 4.4.12. Antioxidant Activities

##### DPPH Radical Scavenging Activity

The DPPH radical scavenging activity of the gel was assessed employing the methodology described by Zheng et al. [101]. The detailed procedure is outlined in the Supplemental Materials.

##### ABTS Radical Scavenging Activity

The ABTS radical scavenging activity of the gels was determined, as described by Zheng et al. [101]. The complete procedure is outlined in the Supplemental Materials.

##### Antimicrobial Activities

The antimicrobial activities of surimi gels were assessed using the methodology described by Roy et al. [102]. The comprehensive procedure is outlined in the Supplemental Materials.

##### Statistical Analysis

The data were subjected to one-way analysis of variance (ANOVA) to determine significant differences in the measured parameters among treatments. Differences between means were resolved using Duncan’s multiple range test (DMRT). Statistical analyses were performed using SPSS (SPSS 23.0 for Windows, IBM, SPSS Inc., Chicago, IL, USA). The data are expressed as mean values ± standard deviations (SDs).

## Figures and Tables

**Table 1:** Effects of different levels of Q on the proximate composition (wet weight basis, %) and pH of CHOS-enriched pangasius surimi gel.

Treatments	Moisture (%)	Protein (%)	Fat (%)	Ash (%)	pH
Control	81.29 ± 0.04 ^d^	12.84 ± 0.01 ^e^	0.77 ± 0.01 ^d^	2.90 ± 0.02 ^b^	6.88 ± 0.02 ^a^
Q1	81.42 ± 0.07 ^bc^	12.61 ± 0.07 ^f^	1.35 ± 0.02 ^a^	2.74 ± 0.02 ^c^	6.78 ± 0.02 ^b^
Q2	81.49 ± 0.03 ^ab^	12.91 ± 0.03 ^d^	0.63 ± 0.01 ^e^	2.88 ± 0.02 ^b^	6.76 ± 0.01 ^b^
Q3	81.53 ± 0.06 ^a^	13.24 ± 0.02 ^c^	0.83 ± 0.01 ^c^	2.93 ± 0.02 ^a^	6.66 ± 0.02 ^c^
Q4	81.29 ± 0.02 ^d^	13.55 ± 0.02 ^b^	0.78 ± 0.01 ^d^	2.90 ± 0.02 ^b^	6.57 ± 0.01 ^d^
Q5	81.40 ± 0.02 ^c^	13.70 ± 0.01 ^a^	0.95 ± 0.01 ^b^	2.89 ± 0.01 ^b^	6.53 ± 0.02 ^e^

Values are expressed as means ± standard deviations (n = 3). Means within a column followed by different letters are significantly different (*p* < 0.05). CHOS-enriched pangasius surimi gel without Q (control); CHOS-enriched pangasius surimi gel with 0.01% Q (Q1); CHOS-enriched pangasius surimi gel with 0.02% Q (Q2); CHOS-enriched pangasius surimi gel with 0.03% Q (Q3); CHOS-enriched pangasius surimi gel with 0.04% Q (Q4); CHOS-enriched pangasius surimi gel with 0.05% Q (Q5).

**Table 2:** Effects of different levels of Q on gel properties of CHOS-enriched pangasius surimi gel.

Treatments	Breaking Force (g)	Deformation (cm)	Gel Strength (g cm)
Control	588.30 ± 21.34 ^d^	0.91 ± 0.02 ^c^	599.68 ± 16.47 ^c^
Q1	612.18 ± 13.08 ^c^	0.99 ± 0.01 ^bc^	613.93 ± 24.39 ^c^
Q2	623.78 ± 11.00 ^bc^	0.99 ± 0.02 ^b^	637.20 ± 11.31 ^b^
Q3	641.66 ± 08.36 ^ab^	1.00 ± 0.02 ^b^	648.31 ± 13.66 ^b^
Q4	654.70 ± 15.20 ^a^	1.13 ± 0.10 ^a^	707.82 ± 15.43 ^a^
Q5	621.12 ± 10.10 ^c^	0.96 ± 0.02 ^bc^	643.83 ± 8.53 ^b^

Values are expressed as means ± standard deviations (n = 5). Means within a column followed by different letters are significantly different (*p* < 0.05). CHOS-enriched pangasius surimi gel without Q (control); CHOS-enriched pangasius surimi gel with 0.01% Q (Q1); CHOS-enriched pangasius surimi gel with 0.02% Q (Q2); CHOS-enriched pangasius surimi gel with 0.03% Q (Q3); CHOS-enriched pangasius surimi gel with 0.04% Q (Q4); CHOS-enriched pangasius surimi gel with 0.05% Q (Q5).

**Table 3:** Effects of different levels of Q on TPA of CHOS-enriched pangasius surimi gel.

Treatments	Hardness (g)	Springiness (cm)	Cohesiveness	Chewiness (g cm)	Resilience
Control	6377.08 ± 232.93 ^e^	0.93 ± 0.00 ^d^	0.81 ± 0.00 ^b^	4524.90 ± 220.34 ^c^	0.49 ± 0.00 ^c^
Q1	6969.57 ± 472.81 ^d^	0.94 ± 0.00 ^c^	0.82 ± 0.00 ^b^	4700.86 ± 129.85 ^c^	0.49 ± 0.01 ^c^
Q2	7417.13 ± 297.76 ^c^	0.95 ± 0.01 ^b^	0.82 ± 0.01 ^b^	5095.38 ± 324.02 ^b^	0.50 ± 0.01 ^bc^
Q3	7814.82 ± 128.88 ^b^	0.95 ± 0.01 ^b^	0.82 ± 0.01 ^b^	5213.09 ± 172.08 ^ab^	0.50 ± 0.01 ^bc^
Q4	8484.51 ± 157.05 ^a^	0.96 ± 0.00 ^a^	0.83 ± 0.01 ^a^	5509.15 ± 379.35 ^a^	0.52 ± 0.01 ^a^
Q5	7440.82 ± 264.27 ^c^	0.95 ± 0.01 ^bc^	0.82 ± 0.00 ^b^	5168.24 ± 265.98 ^ab^	0.49 ± 0.00 ^c^

Values are expressed as means ± standard deviations (n = 5). Means within a column followed by different letters are significantly different (*p* < 0.05). CHOS-enriched Pangasius surimi gel without Q (control); CHOS-enriched Pangasius surimi gel with 0.01% Q (Q1); CHOS-enriched Pangasius surimi gel with 0.02% Q (Q2); CHOS-enriched Pangasius surimi gel with 0.03% Q (Q3); CHOS-enriched Pangasius surimi gel with 0.04% Q (Q4); CHOS-enriched Pangasius surimi gel with 0.05% Q (Q5).

**Table 4:** Effects of different levels of Q on the instrumental colour (L* (lightness), a* (redness/greenness), and b* (yellowness/blueness)) and whiteness index of CHOS-enriched pangasius surimi gel.

Treatments	L* (Lightness)	a* (Redness/Greenness)	b* (Yellowness/Blueness)	Whiteness Index
Control	81.03 ± 0.22 ^a^	0.38 ± 0.02 ^a^	16.35 ± 0.11 ^f^	74.97 ± 0.13 ^a^
Q1	73.70 ± 0.06 ^b^	−0.88 ± 0.02 ^b^	21.53 ± 0.05 ^e^	65.93 ± 0.03 ^b^
Q2	73.32 ± 0.09 ^c^	−1.27 ± 0.00 ^c^	23.59 ± 0.11 ^d^	64.65 ± 0.06 ^c^
Q3	72.74 ± 0.16 ^d^	−1.34 ± 0.02 ^d^	24.76 ± 0.06 ^c^	63.26 ± 0.14 ^d^
Q4	72.33 ± 0.33 ^e^	−1.71 ± 0.01 ^e^	25.52 ± 0.04 ^b^	62.69 ± 0.13 ^e^
Q5	71.91 ± 0.49 ^f^	−2.06 ± 0.02 ^f^	26.73 ± 0.09 ^a^	62.58 ± 0.05 ^e^

Values are expressed as means ± standard deviations (n = 5). Means within a column followed by different letters are significantly different (*p* < 0.05). CHOS-enriched pangasius surimi gel without Q (control); CHOS-enriched pangasius surimi gel with 0.01% Q (Q1); CHOS-enriched pangasius surimi gel with 0.02% Q (Q2); CHOS-enriched pangasius surimi gel with 0.03% Q (Q3); CHOS-enriched pangasius surimi gel with 0.04% Q (Q4); CHOS-enriched pangasius surimi gel with 0.05% Q (Q5).

**Table 5:** Effects of different levels of Q on the TCA soluble peptide content, Ca^2+^ -ATPase activity, and TSC of CHOS-enriched pangasius surimi gel.

Treatments	TCA Soluble Peptide Content (mg/g)	Ca^2+^-ATPase Activity (µmol Pi/mg Protein/min)	Total Sulfhydryl Content (µmol/g)
Control	1.22 ± 0.05 ^a^	1.12 ± 0.01 ^f^	14.80 ± 0.15 ^a^
Q1	1.21 ± 0.02 ^ab^	1.17 ± 0.02 ^e^	14.25 ± 0.44 ^ab^
Q2	1.19 ± 0.02 ^ab^	1.33 ± 0.04 ^c^	13.89 ± 0.22 ^b^
Q3	1.16 ± 0.02 ^bc^	1.41 ± 0.02 ^b^	12.83 ± 0.09 ^c^
Q4	1.08 ± 0.01 ^d^	1.52 ± 0.01 ^a^	11.61 ± 0.62 ^d^
Q5	1.14 ± 0.02 ^c^	1.27 ± 0.02 ^d^	13.13 ± 0.38 ^c^

Values are expressed as means ± standard deviations (n = 3). Means within a column followed by different letters are significantly different (*p* < 0.05). CHOS-enriched pangasius surimi gel without Q (control); CHOS-enriched pangasius surimi gel with 0.01% Q (Q1); CHOS-enriched pangasius surimi gel with 0.02% Q (Q2); CHOS-enriched pangasius surimi gel with 0.03% Q (Q3); CHOS-enriched pangasius surimi gel with 0.04% Q (Q4); CHOS-enriched pangasius surimi gel with 0.05% Q (Q5).

**Table 6:** Effects of different levels of Q on thermal stability properties of CHOS-enriched pangasius surimi sol.

Treatments	Parameter	Peak-I	Peak-II	Peak-III
Control	Peak temperature (°C)	51.70	57.10	93.10
	Enthalpy (J/g)	−23.40	−7.14	−548.44
Q1	Peak temperature (°C)	-	-	95.40
	Enthalpy (J/g)	-	-	−701.81
Q2	Peak temperature (°C)	56.20	78.50	91.90
	Enthalpy (J/g)	−63.09	−21.82	−207.21
Q3	Peak temperature (°C)	65.40	72.00	96.20
	Enthalpy (J/g)	−9.04	−22.87	−184.04
Q4	Peak temperature (°C)	67.70	-	96.00
	Enthalpy (J/g)	−0.09	-	−2075.01
Q5	Peak temperature (°C)	51.60	57.70	92.00
	Enthalpy (J/g)	−107.86	−505.25	−99.99

CHOS-enriched pangasius surimi sol without Q (Control); CHOS-enriched pangasius surimi sol with 0.01% Q (Q1); CHOS-enriched pangasius surimi sol with 0.02% Q (Q2); CHOS-enriched pangasius surimi sol with 0.03% Q (Q3); CHOS-enriched pangasius surimi sol with 0.04% Q (Q4); CHOS-enriched pangasius surimi sol with 0.05% Q (Q5).

## Data Availability

The data used to support the findings of this study can be made available by the corresponding author upon request.

## Supplementary Materials

The following supporting information can be downloaded at: https://www.mdpi.com/article/10.3390/gels12090776/s1, Figure S1: Effect of different levels of Q on the thermal stability properties of CHOS enriched pangasius surimi sol. CHOS enriched pangasius surimi sol without Q (Control; C); CHOS enriched pangasius surimi sol with 0.01 % Q (Q1); CHOS enriched pangasius surimi sol with 0.02 % Q (Q2); CHOS enriched pangasius surimi sol with 0.03 % Q (Q3); CHOS enriched pangasius surimi sol with 0.04 % Q (Q4); CHOS enriched pangasius surimi sol with 0.05 % Q (Q5).

## Abbreviations

The following abbreviations are used in this manuscript:

CHOS	Chitooligosaccharides
Q	Quercetin
GS	Gel strength
DF	Deformation
BF	Breaking force
ABTS	2,2′-azino-bis (3-ethylbenzothiazoline-6-sulfonic acid)
DPPH	2,2-diphenyl-1-picrylhydrazyl
EMC	Expressible moisture content
WHC	Water-holding capacity
TCA	Trichloroacetic acid
TSC	Total sulfhydryl content
SDS-PAGE	Sodium dodecyl sulphate polyacrylamide gel electrophoresis
SEM	Scanning electron microscopy
DSC	Differential scanning calorimetry
