# Supplementary material for: Impact of Quercetin on the Functional and Structural Properties of Chitooligosaccharide (CHOS)-Enriched Surimi
Source: Gels. 2026 Aug 29;12(9):776. doi: 10.3390/gels12090776 (PMC13606280; doi:10.3390/gels12090776)
Supplement: Supplementary file 1 [file gels-12-00776-s001.zip › gels-4490892-supplementary.pdf]

*Article*

# Impact of Quercetin on the Functional and Structural Properties of Chitooligosaccharide (CHOS)-Enriched Surimi

**Bikash Kumar Pati [1,2], M. Bhargavi Priyadarshini [2,*], Naresh Kumar Mehta [2], Arun Bhai Patel [2], Kumar Gaurav [2] and Shreya Anand [2]**

1 College of Fisheries, Odisha University of Agriculture and Technology, Berhampur, Odisha 760007, India; bikashprocessing@gmail.com
2 College of Fisheries, Central Agricultural University, Agartala, Tripura (West) 799210, India; mocherla.bhargavi@gmail.com, nareshfishco@gmail.com, arun.b.patel@gmail.com, kgcof201921@gmail.com, shreyaanand009@gmail.com
* Correspondence: mocherla.bhargavi@gmail.com; Tel.: +918286658313

## Supplementary material

### "Materials and methods"

*"Expressible moisture content (EMC)"*

The EMC of gels was determined by the method adopted by Chanarat et al. [92]. Cylindrical gel samples were cut into a thickness of 5 mm, weighed accurately (X) and placed between three pieces of Whatman paper No. 1 (Maidstone, Kent, England) at the bottom and two pieces on the top of the sample. The standard weight (5 kg) was placed on the samples between the Whatman paper for 2 min. The samples were then removed from the papers and weighed again (Y). With the following equation, the expressible moisture content was measured and expressed as a percentage of sample weight:

$$EMC (\%) = [(X-Y)/X] \times 100$$

*"Water holding capacity (WHC)"*

The WHC of the surimi gels was analysed following the method specified by Kong et al. [93]. The gels (5 g) were precisely weighed ($W_1$), covered in 3 layers of filter paper, and subsequently placed into a 50 mL centrifuge tube. Gels were subjected to centrifugation (Eppendorf Centrifuge 5910 Ri) at 6000 rpm for 15 min at 4 °C. Following centrifugation, gel samples were immediately weighed ($W_2$).
The WHC was measured in the following way:

$$WHC (\%) = W_2/W_1 \times 100$$

*"TCA soluble peptide content"*

The TCA-soluble peptide content was assessed using a procedure outlined by Buamard et al. [94]. A 2 g gel sample was blended with 18 mL of 5 % TCA solution at 9,000 rpm for 3 min by a homogenizer (Remi Lab Stirrer, Model RQ-127D). The mixture was allowed to stand for 60 min at 5 °C and subsequently centrifuged (Eppendorf Centrifuge 5910 Ri) at 7000 rpm for 10 min at room temperature. The Biuret method (Robinson and Hogden; 1940) was used to quantify the amount of TCA-soluble peptide in the supernatant, which was presented as mg of protein per g of sample.

*"$Ca^{2+}$-ATPase activity"*

The ATPase activity was assessed according to the methodology stated by Endoo and Yongsawatdigul [95]. 500 µL of protein solution was mixed with 250 µL of 0.6 M Tris-HCl (pH 7.0) and 250 µL of 0.1 M $CaCl_2$. Distilled water then mixed to the solution, attaining a total volume of 3.75 mL. Subsequently, 250 µL of 20 mM ATP was poured into to the solution and kept for 5 min at 27 °C. The reaction was terminated by the addition of 2.5 mL of cold 15% (w/v) TCA solution. The mixture was immediately filtered, and the inorganic phosphate (Pi) released in the supernatant was quantified using a spectrophotometer (Thermo Scientific Multiskan GO, version 1.00.40, Finland). The absorbance was recorded at 660 nm and activity was presented as µg Pi/mg protein/min.

*"Total sulfhydryl content"*

The total sulfhydryl content of the samples was assessed following the methodology of Lin et al. [96]. 8 mL of buffer solution (0.086 M Tris-HCl, 4 mM EDTA, 8 M urea, 0.09 M Gly, pH 8.0) was added with 1 mL of sample, and the mixture was centrifuged (Eppendorf Centrifuge 5910 Ri) at 3000 rpm for 15 min at 4 °C. Then, 0.5 mL of Ellman reagent (40 mM DTNB, 0.05 M $Na_2HPO_4$, pH 7.0) was mixed with 4.5 mL of the aforesaid supernatant. The sample was placed in the dark at ambient temperature for 30 min before the absorbance readings were obtained at 412 nm using an ultraviolet spectrophotometer (Thermo Scientific Multiskan GO, version 1.00.40, Finland). The total sulfhydryl contents of the samples were obtained according to following equation:

$$C_{SH} = (A \times D)/(C \times B)$$

where $C_{SH}$ denotes the sulfhydryl content (mol/g), A signifies the absorbance of the sample, B indicates the protein concentration, C represents the molar extinction coefficient (13,600 $M^{-1}$ $cm^{-1}$), and D refers to the dilution factor.

*"Molecular interaction"*

The quantification of soluble protein concentration was performed according to the methodology proposed by Liu et al. [97]. The samples (4 g) were homogenized (Remi Lab Stirrer, Model RQ-127D) for 1 min with either 20 mL 0.05 M NaCl (P1), 0.6 M NaCl (P2), 0.6 M NaCl and 1.5 M urea (P3), or 0.6 M NaCl and 8 M urea (P4). The

samples were kept at 4 °C for 1 h and afterward centrifuged (Eppendorf Centrifuge 5910 Ri) at 7000 rpm for 20 min. The protein concentration of the supernatant was assessed using the Biuret method (Robinson and Hogden, 1940). The indices of ionic bonds, hydrogen bonds and hydrophobic interaction were computed as follows:

Index of ionic bonds = P2 - P1,

Index of hydrogen bonds = P3 - P2,

Index of hydrophobic interaction = P4 - P3

*"Protein patterns"*

Protein patterns of surimi gel were observed using SDS-PAGE as per Laemmli [98]. Surimi (3 g) was blended with 27 mL of 5 % (w/v) SDS and homogenized (Remi Lab Stirrer, Model RQ-127D) for 2 min at 10000 rpm. After keeping at 90 °C for 60 min to solubilise the entire protein, the sample was subjected to centrifugation (Eppendorf Centrifuge 5910 Ri) at 8000 rpm for 15 min at room temperature to eliminate the precipitate. The Biuret assay (Robinson and Hogden, 1940) was carried out to determine the protein concentration of the supernatant. Next, the sample buffer was added to ensure that all samples had the uniform protein concentration. The samples, which contained 15 μg of protein, were finally placed onto the SDS-PAGE apparatus (Mini-protein unit, Bio-Rad, USA) while maintaining a continuous current supply of 15 mA. Once the gels were separated, they were stained, destained, and photographed under white light.

*"Scanning electron microscopy (SEM)"*

Following the protocol laid out by Chen et al. [99], the surimi gels' microstructure was studied using SEM. The gels were sliced into pieces of 2-3 mm thick and then dipped in glutaraldehyde solution (3 %) for 24 h at 4 °C for fixation. After rinsing in distilled water for 1 h, the gels were dehydrated in ethanol solutions containing 40, 60, 80, and 100 % (v/v) for 2 h each time. The SEM (Verios G4, EDAX Inc.) operating at 15 kV was used to study the dehydrated samples.

***"****Antioxidant activities****"***

"DPPH radical scavenging activity"

The method described by Zheng et al. [101] was used to determine the DPPH radical scavenging activity of the gel. The samples were added with deionised water at a ratio of 1:20 (v/v) and homogenised (Remi Lab Stirrer, Model RQ-127D) for 15 min. The solutions were then centrifuged (Eppendorf Centrifuge 5910 Ri) at 3000 rpm for 5 min to remove the precipitate. Each 0.8 mL supernatant was blended with 0.8 mL DPPH working solution (0.1 mM), incubated for 40 min at 27 °C in dark, and subsequently, its absorbance was determined at 517 nm. The DPPH scavenging activity was determined using the following formula:

$$\text{DPPH scavenging activity (\%)} = (S_0 - S_1/S_0) \times 100$$

Where, $S_1$ and $S_0$ are the absorbance of the sample and blank (using distilled water instead of sample), respectively.

"ABTS radical scavenging activity"

The method described by Zheng et al. [101] was used to determine the DPPH radical scavenging activity of the gel. The samples were added with deionised water at a ratio of 1:20 (v/v) and homogenised (Remi Lab Stirrer, Model RQ-127D) for 15 min. The solutions were then centrifuged (Eppendorf Centrifuge 5910 Ri) at 3000 rpm for 5 min to remove the precipitate. To prepare an ABTS stock solution, 2.4 mM potassium persulphate solution was added to an equal volume of 7 mM ABTS solution. The mixed solution was incubated at ambient temperature for 12 to 16 h in the dark. Dilute each 2 mL of stock solution with 100 mL of methanol until the absorbance is 0.70 ± 0.02 at 734 nm at the time of utilization. A mixture of 0.1 mL of supernatant and 1 mL of ABTS working solution was prepared and incubated at 27 °C for 60 min in the dark. The sample's absorbance was computed at 734 nm.

ABTS scavenging activity was evaluated as follows:

$$\text{ABTS scavenging rate (\%)} = (S_0 - S_1/S_0) \times 100$$

Where, $S_1$ and $S_0$ are the absorbance of the sample and blank (using distilled water instead of sample), respectively.

"*Antibacterial activity*"

The antibacterial activity of surimi gels was investigated following the methodology described by Roy et al. [102]. Both Gram (+) (*S. aureus*, ATCC 11632) and Gram (-) (*E. coli*, ATCC 4157) bacteria were selected for the assay. Overnight cultured cells of each bacterium were serially diluted (1:100) in their specific culture broth (30 mL) to obtain an optical density ($OD_{600}$) of 0.05. 30 mL of the diluted cell culture of each bacterium was poured to 100 mL flasks containing 5 g of surimi. A flask with bacterial culture without surimi was considered as the control. The samples were subjected to sterilization by 30 min exposure to UV radiation in a laminar flow system. Then, the flasks were incubated at 37 °C with shaking at 160 rpm for overnight. After being incubated, the cultures from each flask and the control were serially diluted in their corresponding growth media. On tryptic soy agar (TSA) plate, 100 μL of diluted cell culture was spread-plated. The plates were incubated at 37 °C for 24 h. After counting the colonies on each plate, the following formula was used to calculate the bacterial growth inhibition:

$$\text{Inhibition (\%)} = (Bc - Bs)/ Bc \times 100$$

Where Bc represents average no. of bacteria colonies of control and Bs represents the average numbers of bacteria colonies of the sample

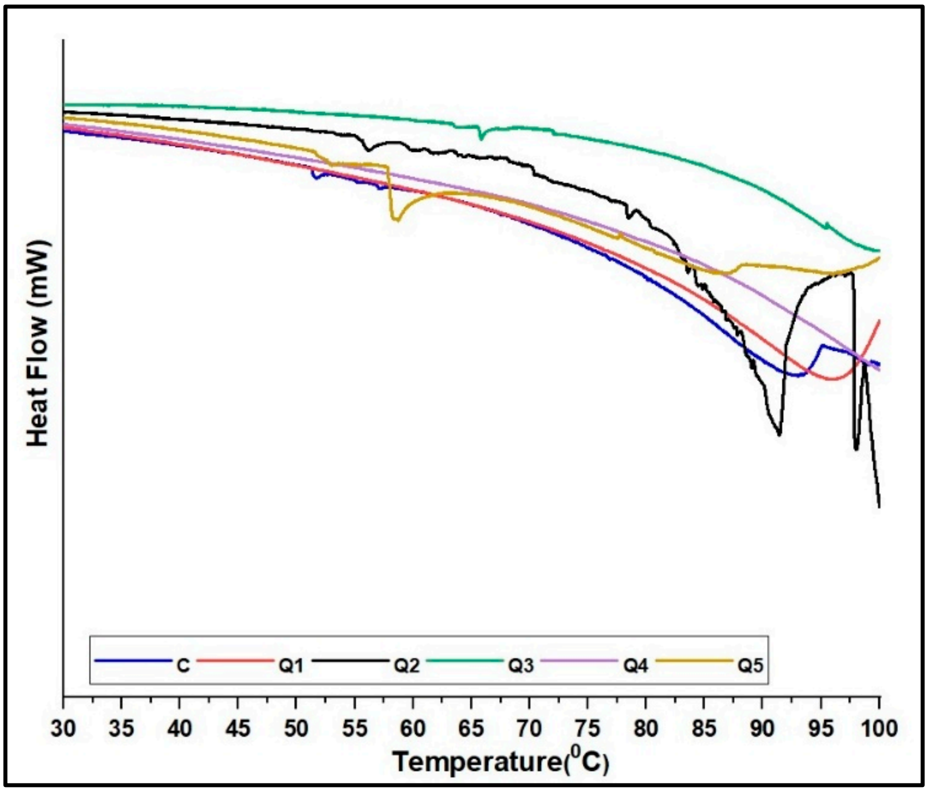


**Figure S1.** Effect of different levels of Q on the thermal stability properties of CHOS enriched pangasius surimi sol. CHOS enriched pangasius surimi sol without Q (Control; C); CHOS enriched pangasius surimi sol with 0.01 % Q (Q1); CHOS enriched pangasius surimi sol with 0.02 % Q (Q2); CHOS enriched pangasius surimi sol with 0.03 % Q (Q3); CHOS enriched pangasius surimi sol with 0.04 % Q (Q4); CHOS enriched pangasius surimi sol with 0.05 % Q (Q5).
